# Environmental and Genetic Factors Associated with Solanesol Accumulation in Potato Leaves

**DOI:** 10.3389/fpls.2016.01263

**Published:** 2016-08-25

**Authors:** Raymond Campbell, Sabine Freitag, Glenn J. Bryan, Derek Stewart, Mark A. Taylor

**Affiliations:** ^1^Cell and Molecular Sciences, The James Hutton InstituteDundee, UK; ^2^Environmental and Biochemical Sciences, The James Hutton InstituteDundee, UK

**Keywords:** solanesol, environment, potato, QTL mapping, isoprenoid, heat stress

## Abstract

Solanesol is a high value 45-carbon, unsaturated, all-trans-nonaprenol isoprenoid. Recently solanesol has received particular attention because of its utility, both in its own right and as a precursor in the production of numerous compounds used in the treatment of disease states. Solanesol is found mainly in solanaceous crops such as potato, tomato, tobacco and pepper where it accumulates in the foliage. There is considerable potential to explore the extraction of solanesol from these sources as a valuable co-product. In this study we have characterized the genetic variation in leaf solanesol content in a biparental, segregating diploid potato population. We demonstrate that potato leaf solanesol content is genetically controlled and identify several quantitative trait loci associated with leaf solanesol content. Transient over-expression of genes from the methylerythritol 4-phosphate (MEP) and mevalonic acid (MVA) pathways, either singly or in combination, resulted in enhanced accumulation of solanesol in leaves of *Nicotiana benthamiana*, providing insights for genetically engineering the pathway. We also demonstrate that in potato, leaf solanesol content is enhanced by up to six-fold on exposure to moderately elevated temperature and show corresponding changes in expression patterns of MEP and MVA genes. Our combined approaches offer new insights into solanesol accumulation and strategies for developing a bio-refinery approach to potato production.

## Introduction

Solanesol is a 45-carbon, all-trans-nonaprenol, first isolated from flue-cured tobacco (Rowland et al., 1956) and subsequently found in the foliage of other solanaceous crops, including potatoes, tomatoes, eggplants, and peppers (Gao et al., 2007; Kotipalli et al., 2008; Ma et al., 2009). In the pharmaceutical industry solanesol is used as an intermediate in the synthesis (both chemically and biotechnologically) of metabolically active quinones such as coenzyme Q10 and vitamin K analogs (Naruta, 1980; Hamamura et al., 2002; Choi et al., 2005; Tian et al., 2010; Athiyaman and Sankaranarayanan, 2014). There is also a growing awareness that solanesol may have useful properties in its own right with reports of anti-bacterial, anti-inflammation, and anti-ulcer activities (Serebryakov and Nigmatov, 1990; Khidyrova and Shakhidoyatov, 2002; Guo et al., 2008; Li and Chase, 2010). Furthermore, other solanesol conjugates such as *N*-solanesyl-*N, N*′-bis (3,4-dimethoxybenzyl) ethylenediamine (SDB- ethylenediamine) and its derivatives (Wang et al., 2007) have received significant attention with respect to their ability to reverse multidrug resistance and sensitize tumor cells to conventional anticancer treatments (Sidorova et al., 2002). Other solanesol derivatives are used in the treatment of cardiovascular disease, osteoporosis, acquired immune deficiency syndrome (AIDS) and wound healing (Srivastava et al., 2009; Hu and Wang, 2011; Yan et al., 2015).

As the foliage of many solanaceous crops such as potato and tomato is essentially a waste product, there is considerable potential to explore the extraction of solanesol from these sources as a valuable co-product. However, the factors that determine the level of solanesol accumulation in these potential biosources are not yet well understood. In tobacco there is considerable genotypic variation in leaf solanesol content, ranging from 0.05 to 3.6% dry weight. Developmental stage (Sheen et al., 1978; Zhao et al., 2007) and environment (Schlotzhauer and Kasperbauer, 1989; Bajda et al., 2005, 2009) are also known to impact on solanesol content. Detailed information about levels of solanesol in other species is lacking although values for unspecified cultivars have been published (Kotipalli et al., 2008, Table 1). As with tobacco, there is considerable variation in the levels of solanesol found in potato foliage, ranging from 1.5% of dry weight (Asahina et al., 1977) to 0.04% per gram fresh weight (Ma et al., 2009). In species outside the Solanaceae there is very little information about solanesol accumulation. Reports indicate that solanesol can be detected in leaf extracts from soybean (Kurisaki et al., 1997) and spinach (Sakaihara et al., 2000) although quantitative data has not been published.

**Table 1:** **Solanesol content in plant tissues**.

**Species**	**Tissue**	**Solanesol content (% dry weight)**	**References**
*Nicotiana tabacum*	Leaf	0.64–0.57	Zhao et al., 2005
*Nicotiana tabacum*	Stalk	0.19–0.06	Zhao et al., 2005
*Nicotiana tabacum*	Flower	0.03	Zhao et al., 2005
*Nicotiana tabacum*	Leaf	0.40–1.70	Zhou and Liu, 2006
*Nicotiana tabacum*	Leaf	0.05–1.77	Kotipalli et al., 2008
*Nicotiana tabacum*	Leaf	0.30–3.0	Chen et al., 2007
*Nicotiana tabacum*	Leaf	1.78–3.6	Yan et al., 2015
*Nicotiana tabacum*	Upper leaf	1.06–1.50	Liu et al., 2007
*Nicotiana tabacum*	Middle leaf	0.41–0.64	Liu et al., 2007
*Nicotiana tabacum*	Lower leaf	0.20 – 0.41	Liu et al., 2007
*Nicotiana tabacum*	Upper peduncle	0.022–0.141	Liu et al., 2007
*Nicotiana tabacum*	Middle peduncle	0.010–0.029	Liu et al., 2007
*Nicotiana tabacum*	Lower peduncle	0–0.011	Liu et al., 2007
*Nicotiana tabacum*	Leaf	0.45	Hu et al., 2007
*Nicotiana tabacum*	Penduncle	0.037	Hu et al., 2007
*Nicotiana tabacum*	Stalk	0.0037	Hu et al., 2007
*Nicotiana tabacum*	Root	0.0013	Hu et al., 2007
*Solanum tuberosum*	Leaf	0.331	Gao et al., 2007
*Solanum tuberosum*	Leaf	1.0–3.0	Asahina et al., 1977
*Solanum tuberosum*	Leaf	0.4	Kotipalli et al., 2008
*Solanum tuberosum*	Leaf	0.04	Ma et al., 2009
*Solanum lycopersicum*	Leaf	0.35	Kotipalli et al., 2008
*Solanum lycopersicum*	Leaf	0.207	Gao et al., 2007
*Solanum melonga*	Leaf	0.4	Kotipalli et al., 2008
*Capsicum annum*	Leaf	0.089	Gao et al., 2007
*Capsicum annum*	Leaf	0.35	Kotipalli et al., 2008
*Datura stramonium*	Leaf	0.15	Kotipalli et al., 2008
*Solanum nigrum*	Leaf	0.25	Kotipalli et al., 2008
*Nicandra physaloides*	Leaf	0.3	Kotipalli et al., 2008
*Cestrum nocturnum*	Leaf	0.05	Kotipalli et al., 2008
*Solanum xanthocarpum*	Leaf	0.25	Kotipalli et al., 2008
*Glycine max*	Leaf	Not specified	Kurisaki et al., 1997
*Spinacea oleracea*	Leaf	Not specified	Sakaihara et al., 2000

The formation of isoprenoids such as solanesol is compartmentalized and under developmental regulation (reviewed in Logan et al., 2000). All isoprenoids are composed of isoprene units derived from the common pre-cursor isopentenyl diphosphate (IPP). Prenyl chains are assembled from the consecutive condensation of IPP and its isomer DMAPP, involving the formation of short chains, such as FPP (farnesyl diphosphate, C15) and GGPP (geranylgeranyl diphosphate, C20), which in turn undergo further elongation in reactions catalyzed by long chain prenyl diphosphate synthases. Recently the plastidic prenyl diphosphate synthase responsible for solanesol biosynthesis from tomato has been identified and characterized (Jones et al., 2013).

Solanesol diphosphate is a key intermediate in the biosynthesis of the prenylquinone, plastoquinone (PQ). PQ is an electron carrier and has numerous cellular functions. In chloroplasts, for example, PQ is a component of the electron-transport chain acting between photosystem II and the cytochrome b_6_f complex. The pathway that leads to PQ has been elucidated (Nowicka and Kruk, 2010) and the isoprene units of solanesol are solely biosynthesized via the MEP pathway which is believed to occur exclusively in the plastid (Rodríguez-Concepción and Boronat, 2002; Fukusaki et al., 2004). Biosynthetically, solanesol is derived from solanesyl diphosphate (SPP) and occurs in both free and esterified forms (Rowland and Latimer, 1959). Solanesyl ester fractions from tobacco yield a series of fatty acids and solanesol when hydrolyzed (Scholtzhauer et al., 1976). Rowland and Latimer (1959) found that solanesol combined with palmitic, linoleic, linolenic, myristic, and oleic acids and two unidentified acids.

No definitive biological functions have been assigned to free polyisoprenoid alcohols or their carboxylic esters; the main accumulated forms, although recent work has made some progress in this respect. In tobacco leaves infection with tobacco mosaic virus (TMV) or *Pseudomonas syringae* pv. *tabaci* resulted in an increase of up to 7-fold in the level of solanesol and significant increases in the levels of other polyprenol alcohols only in a resistant cultivar (*Nicotiana tabacum* cv. Samsun NN; Bajda et al., 2009). In susceptible cultivars no significant increase was measured. Hydrogen peroxide also elicited a significant increase in solanesol content, leading to the suggestion that polyisoprenoid alcohols may have a role in protection against reactive oxygen.

In view of the commercial potential of solanesol as a co-product in potato production, the aim of this study was to establish environmental and genetic factors that impact on solanesol accumulation. The approach of combining quantitative genetics studies with metabolite analysis, genomics and transcriptomics is a powerful means of identifying the causal genes controlling natural variation in metabolite accumulation (reviewed in Soltis and Kliebenstein, 2015). Large effect QTL that are associated with solanesol accumulation were identified and a major effect of moderately elevated temperature on leaf solanesol content was demonstrated. Detailed transcript profiling and identification of candidate genes underlying a major QTL suggest routes for germplasm enhancement with respect to solanesol accumulation.

## Materials and methods

### Plant material and sampling

Potato plants (cv Desiree) were obtained from Science and Advice for Scottish Agriculture, Edinburgh, UK, as virus-free *in vitro* plantlets and were propagated in 90 mm Petri dishes containing MS medium (Murashige and Skoog, 1962) supplemented with 20 g L^−1^ sucrose and 8 g L^−1^ agar at 18 ± 4°C, 16 h light, light intensity 100 μmol m^−2^s^−1^. Four weeks after subculture *in vitro* plantlets were transferred to 12 cm pots containing compost and grown in a glasshouse, under conditions of 16 h light (18°C) and 8 h dark (15°C). Light intensity ranged from 400 to 1000 μmol m^−2^s^−1^. After 7 weeks, glasshouse-grown plants were subdivided to into two sets and moved to growth rooms under conditions of either 16 h light (22°C, 80% humidity) and 8 h dark (16°C, 70% humidity) or 16 h light (30°C, 80% humidity) and 8 h dark (20°C, 70% humidity). Both rooms had a light intensity of 300 μmol m^−2^s^−1^ and plants were watered twice daily.

Plants used for time course experiments were acclimated in a controlled environment room for 1 week at 22/16°C before a subset was moved to a 30/20°C controlled environment room. Leaf samples from triplicate biological replicates were harvested from the fourth node from the apex of the plant at 1, 24, 48, 72, 96, and 168 h time points. Leaf samples taken from plants acclimated for 1 week at the two growth room temperatures were harvested every 4 h for 24 h from three replicate plants.

### 06H1 population

The bi-parental diploid potato mapping population used in this study (06H1) has previously been described by Prashar et al. (2014). Field trials were carried out as 10 plant plots per genotype, grown in a randomized block design between 2014 and 2015 at Balruddery Farm, near Invergowrie, Dundee. Seed tubers of the 186 clones and the two parents were planted in the last week of April, 30 cm apart in pre-made drills with 2 m distance between plots. All field trials went through standard agronomic practices for fertilizer and pesticide applications. Leaf samples were collected at 12 weeks post emergence (first developmental stage) where the plants were approaching maturity and a second harvest 4 weeks later (second developmental stage). Leaves were taken from the fourth node from the apex of the plant and flash frozen in liquid nitrogen.

### Extraction and quantification of solanesol and photosynthetic pigments

#### Chlorophyll

Leaf samples were harvested into liquid N_2_ and lyophilized in the dark. Dried leaf material was ground under reduced light and extracted in 50 volumes 80% acetone with end-over-end mixing for 1 h in the dark at 4°C. The content of chlorophyll a, chlorophyll b and total carotenoids were estimated by differential spectrometry in the extract according to the method of Lichtenthaler and Wellburn (1983).

#### Solanesol

Aliquots of 20 mg of freeze dried ground potato leaf tissue were weighed into 2 ml Eppendorf tubes. To this, 500 μl ethanol, 250 μl of dH2O (distilled water) and 6% (v/v) KOH were added and the extracts vortexed for 1 min then incubated at 60°C in a shaking incubator at 200 rpm for 4 h to facilitate the extraction and hydrolysis of the fatty acid bound solanesol. Following cooling for 5 min at room temperature, 500 μl of n-hexane was added to the mixture (liquid—liquid extraction) and the samples vortexed for 1 min then ultrasonically extracted using a sonicated bath (Elma, Elmasonic P70H) at 37khz, 100% power for 20 min, inverting the samples every 2 min, prior to centrifugation at 16,000 g for 1 min. The upper non-polar phase was then removed to a fresh 2 ml Eppendorf tube using a pipette and the ultrasonic extraction process repeated a further two times using 500 μl of n-hexane. Recovery experiments demonstrated that this procedure was effective for solanesol extraction from leaf samples, In these experiments up to 96.1% of the processed solanesol standard was recovered from the hexane extract (Figure S1). The combined extracts were then evaporated to dryness using a GeneVac miVac Duo concentrator (GeneVac Ltd., Ipswich UK) at 40°C or under a stream of oxygen free nitrogen gas. The dried extract was then re-constituted in 1 ml of methanol then an aliquot further diluted 1:10 with methanol and filtered using a 0.45 μM PTFE filter vial (Thompson, California) prior to LC-MS analysis.

#### LC-MS analysis quantification of solanesol

Quantification of solanesol was carried out using an Agilent high performance liquid chromatography (HPLC) system (Infinity 1260) equipped with a quaternary pump, DAD, column temperature control department and thermostat coupled to an Agilent 6460A Triple Quadrupole Mass Spectrometer (Agilent Technologies, Santa Clara, USA). Chromatographic separation was achieved by injecting 3 μl of sample onto a reverse phased C30 3 μm column [100 × 4.6 mm] coupled to a 3 μm, 50 × 4.6 mm C30 guard column (YMC Inc., USA), maintained at 25°C. Mobile phases consisted of (A) methanol containing 0.1% formic acid (FA), water/methanol (20/80, v/v) containing 0.2% ammonium acetate (B) and tert-methyl butyl ether containing 0.1% FA. The gradient elution used with this column at a flow rate of 0.4 ml min^−1^ was 95% A, 5% B held isocratically for 2.4 min then stepped up to 80% A, 5% B, 15% C at 2.6 min, followed by a linear gradient to 30% A, 5% B, 65% C by 5 min and held for 2 min. A conditioning phase from 10 to 13 min returned the column to the initial concentrations of A & B.

The mass spectrometer was operated using a jet stream electrospray ionization interface (ESI) in positive ion mode. For optimal ESI settings, drying gas temperature, drying gas flow, nebulizer pressure, sheath gas temperature, sheath gas flow, capillary cap voltage, and nozzle voltage were set to 300°C, 5 L min^−1^, 45 psi, 250°C, 11 L min^−1^, 4.5 kV, and 500 V, respectively. Collision energies and fragmentor voltages for transition states for the standard compounds solanesol and *trans*-β-Apo-8′carotenal (internal standard) were optimized by direct infusion (1 mg L^−1^), resulting in 60V/208V for solanesol and 72V/132 V for *trans*-β-Apo-8′carotenal, respectively. Hereby the most sensitive transitions, i.e., transitions with the highest intensity of the product ion were chosen.

Multiple reaction monitoring (MRM) was used for quantitation of solanesol with the precursor ion of *m/z* 613.6 [M-H_2_O+H]^+^ and the product ion of 81.1 where *trans*-β-Apo-8′carotenal was used as internal standard with precursor ion m/z 417.3 [M+H]^+^ and product ion 91.1 Concentrations of solanesol were calculated using a 6 point calibration curve ranging from 0.1 to 10 mg L^−1^. Calibration curves were established by creating response ratios normalizing the solanesol peak area of the varying concentrations to the peak area of the internal standard *trans*-β-Apo-8′carotenal. Linear range, limit of detection (LOD) and limit of quantification (LOQ) were determined for Solanesol, resulting in 0,1–30 mg L^−1^, 5.2 μg L^−1^, and 17.2 μg L^−1^. Data were collected and analyzed using the Agilent MassHunter Acquisition and Quantitative software (Version B.06.00).

### Microarray/RNA-seq

The data set used in this study has been published by Hancock et al. (2014) and the experimental design and complete datasets are available at ArrayExpress (http://www.ebi.ac.uk/arrayexpress/; accession E-MTAB-1655).

### Construction of the 06H1 linkage map and QTL analysis

The linkage map used in this study consists 2157 mapped single nucleotide polymorphism (SNP) markers comprising 1355 distinct genetic, 802 “co-segregating” markers (Prashar et al., 2014). QTL analysis was performed using MapQTL version 5.0 (Van Ooijen, 2004). An initial rapid search for associations between markers and traits was performed using the non-parametric Kruskal-Wallis (KW) test. QTL were identified by MQM mapping and co-factors identified using the automated co-factor selection tool at a *P*-value of < 0.02. Logarithm of odds (LOD) thresholds for declaring QTL significance were determined by performing 1000 permutations on each individual trait (Churchill and Doerge, 1994).

### Production of greening tubers

Potato tubers from cv. Desiree were left on a laboratory bench exposed to daylight for a period of 28 days, tubers were turned every few days to ensure even light exposure. A set of control tubers were stored in complete darkness in the same location. The room temperature was maintained at 21°C.

### Vector construction

Constructs used for the transient expression of various Methylerythritol 4-phosphate pathway (MEP) genes in *N. benthamiana* were constructed using the GoldenBraid 2.0 modular assembly system (Sarrion-Perdigones et al., 2011). Transcript sequences for MEP pathway genes were obtained from the Potato Genome resource SpudDB database and a list of transcript numbers and primer sequences used for cloning are detailed in Table S1. Genes were cloned and the final constructs assembled according to the cloning protocols generated using the online tool domestication and TU assembler tool on the GoldenBraid website (www.gbcloning.org). Briefly, GBparts and GBpatches were obtained by PCR amplification using Phusion High-Fidelity DNA Polymerase (ThermoScientific) and cDNA prepared from leaf and tuber RNA isolated from the *Solanum tuberosum* cv. Desiree. PCR products were purified using the QIAquick PCR Purification Kit (Qiagen) and quantified using a Nano Drop Spectrophotometer 2000. Then, 40 ng of each amplicon and 75 ng of the domestication vector (pUPD) were mixed and incubated in a BsmBI restriction-ligation reaction. Following incubation of 25 cycles of 37°C for 2 min and 16°C for 5 min, 1 μl of the ligation reaction was transformed into DH5α. Positive clones were selected on ampicillin, 5-bromo-4-chloro-3-indolyl-b-D-galactopyranoside acid (IPTG), and isopropylthio-b-galactoside (X-GAL) containing plates. The correct assembly was confirmed by restriction analyses and sequencing.

For construction of the final binary construct 75 ng of GBpart CaMV 35S promoter (GB0030) and 75 ng of *Agrobacterium tumefaciens* terminator pTnos (GB0037) were mixed with 75 ng of the gene of interest and 75 ng of the pDGB1α1 destination vector in a BSAI restriction ligation. The reaction was incubated and transformed as described in the previous ligation step and the positive clones selected on spectinomycin, IPTG, X-GAL plates. Vector maps of the final constructs used in this study are presented in Figure S2.

### Transient expression in *N. benthamiana*

For the transient expression experiments, plasmids were transferred to *A. tumefaciens* strain AGL1 by electroporation. Agroinfiltration was performed as described previously (Wydro et al., 2006). Overnight-grown bacterial cultures were pelleted and re-suspended in agroinfiltration medium (10 mM MES, pH 5.6, 10 mM MgCl2, and 200 mM acetosyringone) to an optical density at 600 nm of 0.5. Infiltrations were carried out using a needle-free syringe in leaves 2, 3, and 4 of 4- to 5-week-old *N. benthamiana* plants (growing conditions: 24°C day/20°C night in a 16-h-light/8-h-dark cycle). Leaves were harvested 3, 5, and 7 days post infiltration and examined for transgene expression.

## Results

### Genetic variability in potato leaf solanesol content

**Figure 1:**
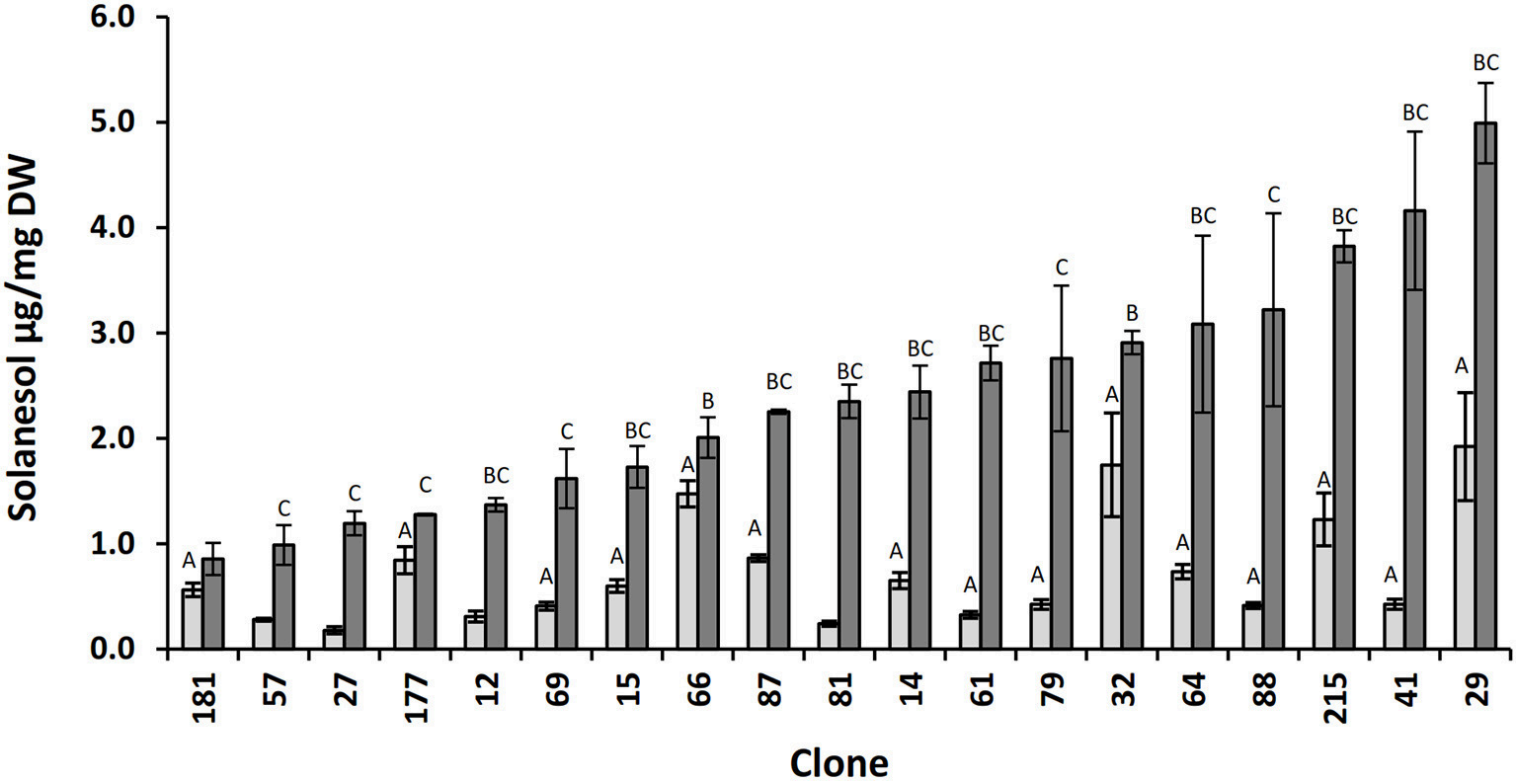
**Relative concentrations of solanesol present in leaf samples of selected 06H1 genotypes**. Comparison of leaf solanesol concentrations (μg/mg DW) in selected 06H1 genotypes measured at two developmental stages. Bars (light grey, first developmental stage; dark grey, second developmental stage) represent the mean value of three biological replicates. Error bars indicate the standard error. Statistical analysis was performed using one way ANOVA and Student's *t*-test (*P* < 0.05) and are indicated by (A) vs. lowest solanesol first developmental stage clone, 181 (B) vs. lowest solanesol second developmental stage clone, 181 (C) first vs. second developmental stage.

Previous reports indicate that in tobacco there is significant genetic variability in leaf solanesol content (Zhao et al., 2005; Hu et al., 2007; Liu et al., 2007). In this study the variation in potato leaf solanesol content was investigated. For this purpose, a bi-parental potato diploid population (06H1) with a previously constructed dense SNP marker linkage map was employed (Prashar et al., 2014). Initial studies assessed likely variation in leaf solanesol content in this population. For this reason, leaf solanesol content was measured in a sub-set of 19 genotypes selected at random from the 06H1 population at two developmental stages. The first stage was harvested 12 weeks post emergence where the plants were approaching maturity and the second harvest 4 weeks later. Leaf solanesol was shown to vary ca. 10-fold (0.18–1.92 μg/g DW) between the selected genotypes in the first developmental stage samples (Figure 1). At this stage, it was also observed that solanesol level depended on the stage at which leaves were harvested. Generally, there was a large (up to 9-fold) increase in solanesol content at the second harvest date, although the degree of this increase was variable (ranging from 0.3-fold for genotype 66 to 9 -fold for genotypes 81 and 41, Figure 1).

As there was a large variation in solanesol content in the sub-set of genotypes examined, a wider analysis of 178 genotypes from the 06H1 population was conducted. Leaf solanesol levels in genotypes grown in 2014 and harvested at the first developmental stage ranged from 0.08 to 1.92 μg/mg DW with a median value of 0.42 μg/mg DW compared with 0.02–2.57 μg/mg DW and a median value of 0.52 μg/mg DW at the same developmental stage in 2015 samples. In extracts from the second developmental stage, solanesol content ranged from 0.30 to 4.99 μg/mg DW in the 2014 season with a median value of 2.19 μg/mg DW compared with a range of 0.29–7.69 μg/mg DW and a median value of 2.05 in the 2015 trial. “Broad-sense” heritabilities were estimated for leaf solanesol content for both developmental stages and seasons as 0.616 (2014, stage one), 0.598 (2014, stage two), 0.664 (2015, stage one), 0.864 (2015, stage two) suggesting a moderately high level of genetic control for the four traits. The complete dataset is available in Table S2.

QTL analysis was conducted for solanesol content for each developmental stage and season. The list of all QTL identified is presented in Table 2. In summary, three QTL were detected in the 2014 first developmental stage data, two of which were present on chromosome 7 at 45.48 and 64.34 cM accounting for 12.4 and 13.3% of the variation respectively. QTL present in the same regions on chromosome 7 were also detected in the second developmental stage data but were absent or declared non-significant in the 2015 season dataset. In addition, QTL present on chromosome 2 were detected in both 2014 and 2015 s developmental stage data that explained 8.8 and 8.2% of the total variation respectively.

**Table 2:** **Summary of the QTL affecting leaf solanesol content in the 06H1 population measured in two growing seasons**.

**Year**	**Stage**	**Mapping**	**LG**	**Position**	**Locus**	**LOD**	***R*^2^(%)**	**Co-factor**	**PT Test**
2014	1	R-MQM	6	0.07	c2_30596	3.60	8.7	C2_28849	3.1
2014	1	R-MQM	7	64.34	c2_28849	5.44	13.3	C2_43963	3.1
2014	1	R-MQM	7	45.48	c1_9878	5.19	12.4	C2_43963	3.1
2014	2	R-MQM	2	30.51	c2_17921	3.82	8.8	C2_40635	3.2
2014	2	R-MQM	7	64.34	c2_28849	4.33	11.0	C2_49495	3.2
2014	2	R-MQM	7	48.95	c2_42761	3.61	8.9	C2_49495	3.2
2015	1	MQM	1	20.96	c2_38726	3.59	9.3	–	3.3
2015	2	MQM	2	30.51	c2_17921	3.19	8.2	–	3.1
2015	2	MQM	11	23.31	c2_57107	3.47	9.1	–	3.1

Abbreviations: R-MQM, restricted multiple QTL mapping; MQM, multiple QTL mapping; LG, linkage group; R^2^(%), percentage of variation explained; PT Test, permutation test.

### Environmental effects

Several QTL were identified for solanesol content and the estimates of broad sense heritability indicated a genetic component was having a moderate influence on these levels. However the correlations between solanesol levels between the two seasons were weak (R values of 0.25 for stage 1 and 0.17 for stage 2) although statistically significant (Table 3). These values suggest there is an additional, relatively strong environmental influence on the traits perhaps explaining the lack of complete correspondence in the QTL detected for the four datasets.

**Table 3:** **Correlation between leaf solanesol content in population 06H1 in data from two growing seasons**.

	**2014_stg1**	**2014_stg2**	**2015_stg1**
2014_stg1	–	–	–
2014_stg2	0.433	–	–
2015_stg1	0.245	0.141	–
2015_stg2	0.201	0.172	0.326

Abbreviations: stg1, stage 1; stg2, stage 2.

Inspection of meteorological records for the two seasons indicated that in 2014 the mean air maximum and the mean air minimum temperatures were both higher for the months of May, June and July compared with the same months in 2015 (Table 4). For example, the mean air maximum was 3.4°C higher in July 2014 compared with the 2015 data and the mean air minimum was 2.3°C cooler in June 2015 compared with the same month in 2014. In addition, the total rainfall recorded over the 4 month period was similar in both years (336.9 and 304.7 mm). However, May 2015 received over half the rainfall compared with the previous year, 35.0 and 76.4 mm respectively.

**Table 4:** **Meteorological records for Dundee (56.41620° N, 2.9707° W) between May and August 2014 and 2015**.

	**Month**	**Mean Air Max °C**	**Monthly Max Air °C**	**Mean Air Min °C**	**Monthly Min Air °C**	**Total Rain mm**	**Total Solar Rad. MJ/m2**
2014	May	15.6	20.5	7.2	1.9	51.3	480.42
	June	18.8	25.8	10.3	6.8	76.4	505.10
	July	21.2	26.0	11.2	6.9	81.8	559.56
	August	18.3	21.0	9.1	4.2	127.4	427.65
2015	May	14.1	18.3	4.6	−1.1	86.0	523.48
	June	17.2	23.2	8.0	2.7	35.0	516.69
	July	17.8	24.0	9.9	5.5	109.7	477.65
	August	19.3	21.7	10.0	5.8	74.0	429.77

**Figure 2:**
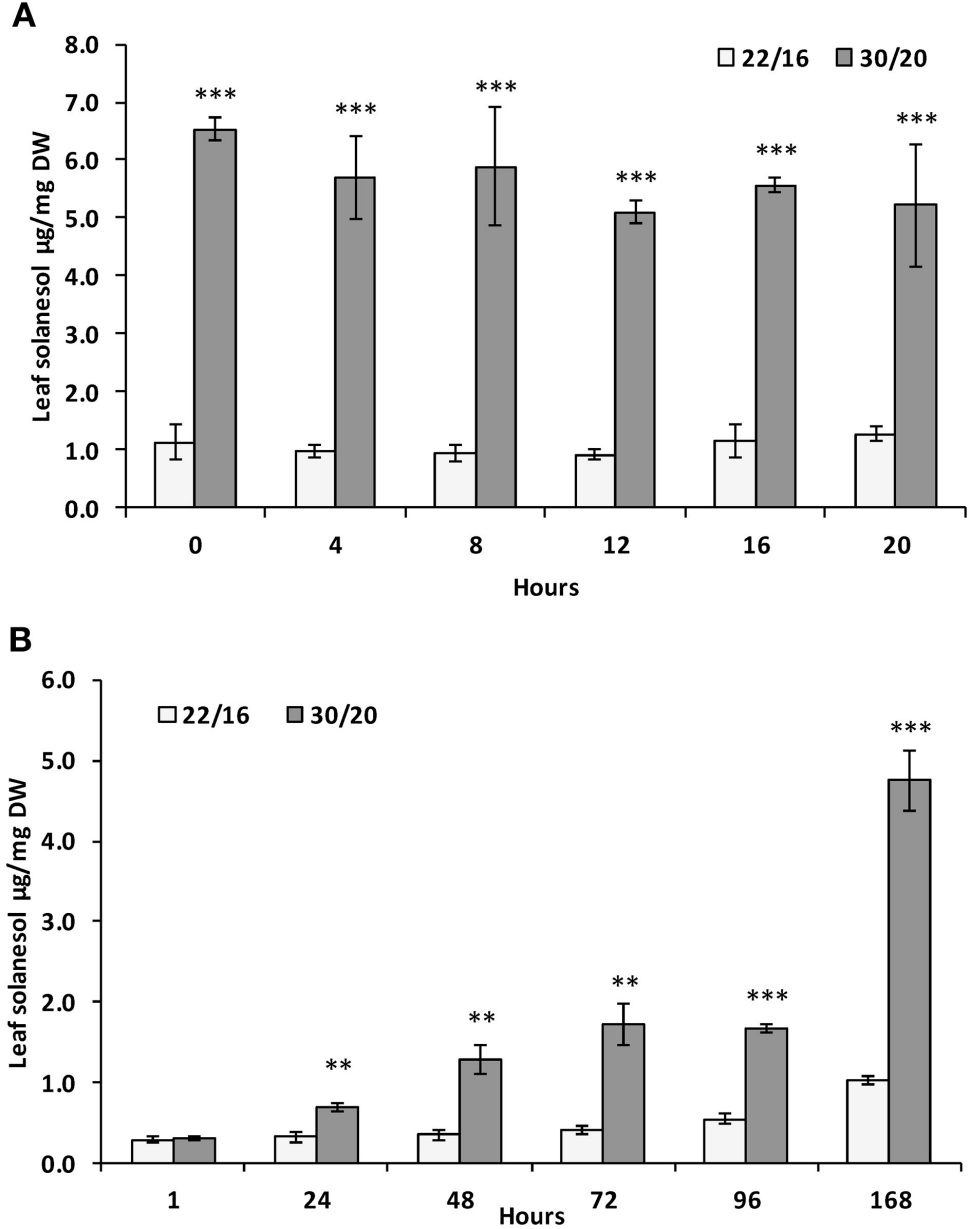
**Relative concentrations of solanesol present in leaf samples exposed to elevated temperature**. Comparison of leaf solanesol concentrations (μg/mg DW) in **(A)** Desiree plants acclimatized for 1 week at either 22/16°C or 30/20°C then sampled every 4 h over a 24 h period and **(B)** Desiree plants acclimatized for 1 week at 22/16°C then moved to 30/20°C and sampled at daily time-points over a period of 1 week. Bars represent the mean value of three biological replicates. Error bars indicate the standard error. Statistical analysis was performed using Student's *t*-test and significance of differences are indicated (^**^P < 0.01, ^***^P < 0.001).

For these reasons, the effects of environmental parameters on leaf solanesol content were investigated under carefully controlled growth conditions. The effects of mildly elevated temperature on leaf solanesol content were investigated in the tetraploid variety Desiree. A temperature regime of 30°C/20°C day/night was selected, which although warmer than the field conditions experienced in this study, are conditions commonly encountered in many major potato growing regions. Furthermore, previous studies have characterized gene expression, metabolite and physiological parameters under these conditions (Hancock et al., 2014). Plants grown at elevated temperatures (30°/20°C day/night) exhibited a shift in leaf solanesol content compared with plants grown under lower temperatures (22°/16°C day/night; Figure 2A). After 1 week under the higher temperature regime, acclimated plants exhibited a ca. 6-fold increase in leaf solanesol content. Leaves were sampled every 4 h over a 24 h period and the solanesol content remained constant at both temperatures (Figure 2A). For plants transferred from 22°/16°C day/night conditions to 30°/20°C day/night, the increase in leaf solanesol content was monitored over seven days as this time period was sufficient to elicit a large increase in solanesol content in acclimated plants (Figure 2A). Significant increases in solanesol content were detected at the elevated temperatures after 24 h and after 7 days where solanesol content was ca. 5-fold higher (Figure 2B).

Transcriptomic datasets for plants grown under identical elevated temperature conditions were available from a previous study (Hancock et al., 2014). From these datasets the normalized expression profiles of genes likely to be involved in solanesol biosynthesis from the MEP and mevalonate (MVA) pathways were examined. Expression profiles which exhibited more than 30% difference in normalized expression values between the two temperature regimes, with a *P* < 0.05, were considered significant. Transcript levels of the MEP pathway genes, 1-deoxy-D-xylulose-5-phosphate synthase 1 (*DXS1*), 1-deoxy-D-xylulose-5-phosphate synthase 2 (*DXS2*), 1-deoxy-D-xylulose-5-phosphate reductoisomerase (*DXR*), 4-hydroxy-3-methylbut-2-en-1-yl diphosphate synthase (*HDS*), and geranylgeranyl pyrophosphate synthetase 2 (*GGPS2*) were all significantly upregulated at the higher temperature (by between 32 and 108% of the normalized expression values detected at the lower temperature). Four MEP pathway genes were downregulated at elevated temperature, 4-diphosphocytidyl-2-C-methyl-D-erythritol kinase (*CMK*), 2-C-methyl-D-erythritol 2,4-cyclodiphosphate synthase (*MCS*), isopentenyl-diphosphate delta-isomerase (*IDI*) and geranylgeranyl pyrophosphate synthase 4 (*GGPS4*) (Table 5A).

**Table 5A:** **Summary of differentially expressed MEP pathway genes identified through microarray analysis in Desiree plants grown at two different temperature regimes**.

**Gene**	**Microarray ID**	**Transcript number**	**Chr**.	**22/16°C norm exp. *n* = 16**	**SE**	**30/20°C norm exp. *n* = 16**	**SE**	**% difference 30/20 vs. 22/16°C**	***P*-Value**
1-deoxy-D-xylulose-5-phosphate synthase 1	CUST_33054_PI426222305	PGSC0003DMT400058821	1	0.85	0.10	1.64	0.27	94.11	^**^
1-deoxy-D-xylulose-5-phosphate synthase 2	CUST_26081_PI426222305	PGSC0003DMT400041591	11	0.90	0.11	1.87	0.26	107.91	^***^
1-deoxy-D-xylulose 5-phosphate reductoisomerase	CUST_12968_PI426222305	PGSC0003DMT400062966	3	0.89	0.04	1.18	0.12	32.29	^*^
4-hydroxy-3-methylbut-2-en-1-yl diphosphate synthase	CUST_4428_PI426222305	PGSC0003DMT400020794	11	0.76	0.06	1.13	0.07	49.99	^***^
Geranylgeranyl pyrophosphate synthase 2	CUST_50887_PI426222305	PGSC0003DMT400071599	4	0.94	0.10	1.51	0.21	59.85	^***^
4-diphosphocytidyl-2-C-methyl-D-erythritol kinase	CUST_42882_PI426222305	PGSC0003DMT400058463	1	1.15	0.11	0.72	0.10	−36.95	^*^
2-C-methyl-D-erythritol 2,4-cyclodiphosphate synthase	CUST_10751_PI426222305	PGSC0003DMT400031768	8	1.51	0.15	0.78	0.05	−48.36	^***^
Isopentenyl-diphosphate Delta-isomerase	CUST_14186_PI426222305	PGSC0003DMT400060039	5	1.27	0.13	0.76	0.08	−40.55	^**^
Geranylgeranyl pyrophosphate synthase 4	CUST_5859_PI426222305	PGSC0003DMT400006940	9	1.27	0.07	0.83	0.06	−34.50	^***^

The data were generated by Hancock et al. (2014) and the values shown are the mean normalized expression values of 16 replicates ± standard error. Comparison of the 22/16° and 30/20°C temperature regimes is shown as a percentage difference. Statistical analysis was performed using Student t-test and significance of differences are indicated (

* P < 0.05,

** P < 0.01,

*** P < 0.001).

Abbreviations: Chr, chromosome.

For genes involved in the MVA pathway, undecaprenyl pyrophosphate synthase (*UPPs*), farnesyl diphosphate synthase (*FPS*), and 3 3-hydroxy-3-methylglutaryl-coenzyme A reductase genes were all upregulated at elevated temperatures increasing by between 57.1 and 113.4% of the normalized expression values detected in the lower temperature plants. Down regulated genes included a 3-hydroxy-3-methylglutaryl coenzyme A reductase, 3-hydroxy-3-methylglutaryl coenzyme A synthase and two mevalonate disphosphate decarboxylase genes (Table 5B).

**Table 5B:** **Summary of differentially expressed MVA pathway genes identified through microarray analysis in Desiree plants grown at two different temperature regimes**.

**Gene**	**Microarray ID**	**Transcript number**	**Chr**.	**22/16°C norm exp. *n* = 16**	**SE**	**30/20°C norm exp. *n* = 16**	**SE**	**% difference**	***P*-Value**
Undecaprenyl pyrophosphate synthetase	CUST_2293_PI426222305	PGSC0003DMT400028817	10	2.24	0.66	3.58	1.11	59.91	^*^
Dimethylallyltransferase	CUST_25558_PI426222305	PGSC0003DMT400037239	10	0.62	0.09	1.20	0.09	92.65	^***^
Farnesyl diphosphate synthase	CUST_25706_PI426222305	PGSC0003DMT400022417	10	0.84	0.03	1.22	0.04	45.35	^***^
3-hydroxy-3-methylglutaryl-coenzyme A reductase 1	CUST_44206_PI426222305	PGSC0003DMT400035541	2	0.79	0.11	1.69	0.23	113.36	^**^
3-hydroxy-3-methylglutaryl-coenzyme A reductase 1	CUST_5012_PI426222305	PGSC0003DMT400092714	11	0.83	0.11	1.30	0.16	57.11	^**^
HMGR CoA reductase	CUST_6140_PI426222305	PGSC0003DMT400095682	11	0.69	0.11	1.19	0.08	72.47	^*^
Cytosolic acetoacetyl-coenzyme A thiolase	CUST_34235_PI426222305	PGSC0003DMT400044777	7	1.42	0.19	0.88	0.08	−38.33	^*^
3-hydroxy-3-methylglutaryl coenzyme A reductase	CUST_7635_PI426222305	PGSC0003DMT400025701	4	1.61	0.06	0.80	0.22	−50.32	^***^
3-hydroxy-3-methylglutaryl coenzyme A reductase	CUST_21783_PI426222305	PGSC0003DMT400008902	2	1.49	0.15	0.91	0.07	−39.05	^**^
3-hydroxy-3-methylglutaryl coenzyme A synthase	CUST_35437_PI426222305	PGSC0003DMT400032619	8	1.37	0.28	0.75	0.19	−44.81	^***^
Mevalonate disphosphate decarboxylase	CUST_50440_PI426222305	PGSC0003DMT400031130	4	1.32	0.06	0.90	0.06	−31.86	^**^
Mevalonate disphosphate decarboxylase	CUST_40387_PI426222305	PGSC0003DMT400061600	11	1.35	0.13	0.92	0.08	−31.71	^**^

The data were generated by Hancock et al. (2014) and the values shown are the mean normalized expression values of 16 replicates ± standard error. Comparison of the 22/16° and 30/20°C temperature regimes is shown as a percentage difference. Statistical analysis was performed using Student t-test and significance of differences are indicated (

* P < 0.05,

** P < 0.01,

*** P < 0.001).

Abbreviations: Chr, chromosome.

Transcripts involved in carotenoid biosynthesis were also differentially expressed at elevated temperatures, where significant upregulation in 2 β carotene hydroxylase genes (*CHY1* and *CHY2*), phytoene synthase 2 (*PSY2*), lycopene β cyclase (*LCY-B*), phytoene desaturase (*PDS*) and violaxanthin de-epoxidase (*VDE*) was observed (Table 5C).

**Table 5C:** **Summary of differentially expressed carotenoid pathway genes identified through microarray analysis in Desiree plants grown at two different temperature regimes**.

**Gene**	**Microarray ID**	**Transcript number**	**Chr**.	**22/16°C norm exp. *n* = 16**	**SE**	**30/20°C norm exp. *n* = 16**	**SE**	**% increase 30/20 vs. 22/16°C**	***P*-Value**
Beta-carotene hydroxylase 2	CUST_42340_PI426222305	PGSC0003DMT400024575	3	0.92	0.19	1.64	0.16	78.25	^**^
Beta-carotene hydroxylase 1	CUST_38469_PI426222305	PGSC0003DMT400074346	6	1.10	0.08	1.52	0.16	38.08	^*^
Lycopene beta cyclase	CUST_45281_PI426222305	PGSC0003DMT400027593	4	0.95	0.06	1.24	0.08	30.07	^***^
Phytoene dehydrogenase	CUST_9305_PI426222305	PGSC0003DMT400023665	3	0.85	0.05	1.21	0.11	42.32	^**^
Phytoene synthase 2	CUST_18568_PI426222305	PGSC0003DMT400043103	3	0.77	0.10	1.39	0.44	80.28	^***^
Violaxanthin de-epoxidase	CUST_4692_PI426222305	PGSC0003DMT400027760	4	0.82	0.08	1.17	0.10	41.63	^***^
Violaxanthin de-epoxidase related	CUST_50238_PI426222305	PGSC0003DMT400080158	4	1.41	0.07	0.75	0.10	−47.08	^***^

The data were generated by Hancock et al. (2014) and the values shown are the mean normalized expression values of 16 replicates ± standard error. Comparison of the 22/16° and 30/20°C temperature regimes is shown as a percentage difference. Statistical analysis was performed using Student t-test and significance of differences are indicated (

* P < 0.05,

** P < 0.01,

*** P < 0.001).

Abbreviations: Chr, chromosome.

A list of differentially expressed transcripts from the Hancock et al. (2014) dataset with a genetic location within 5 Mb of the most significant marker representing QTL for leaf solanesol content in population 06H1 (Table 2) are presented in Table S3. The identified transcripts are thought to be unrelated to the MEP, MVA, and carotenoid pathways. Additional transcriptomic data for various potato tissues sampled under different biotic and abiotic stress conditions is also available from the Spud DB database (Hirsch et al., 2014., http://solanaceae.plantbiology.msu.edu/) where the data provides further insights into MEP pathway gene expression (Table S4). In potato whole plants subjected to elevated temperatures of 35°C for 24 h *DXS1, DXR, CMS, CMK, MCS, IDI*, and *GGPPS3* transcripts are all up-regulated ca. 2-fold at 35°C whereas *SDS* is up-regulated by ca. 4-fold. SDS is also differentially expressed both in wounded and in *Phytophthora infestans* challenged leaves by ca. 12-fold and 2-fold respectively (Table S4).

### Effects of tobacco mosaic virus exposure

**Figure 3:**
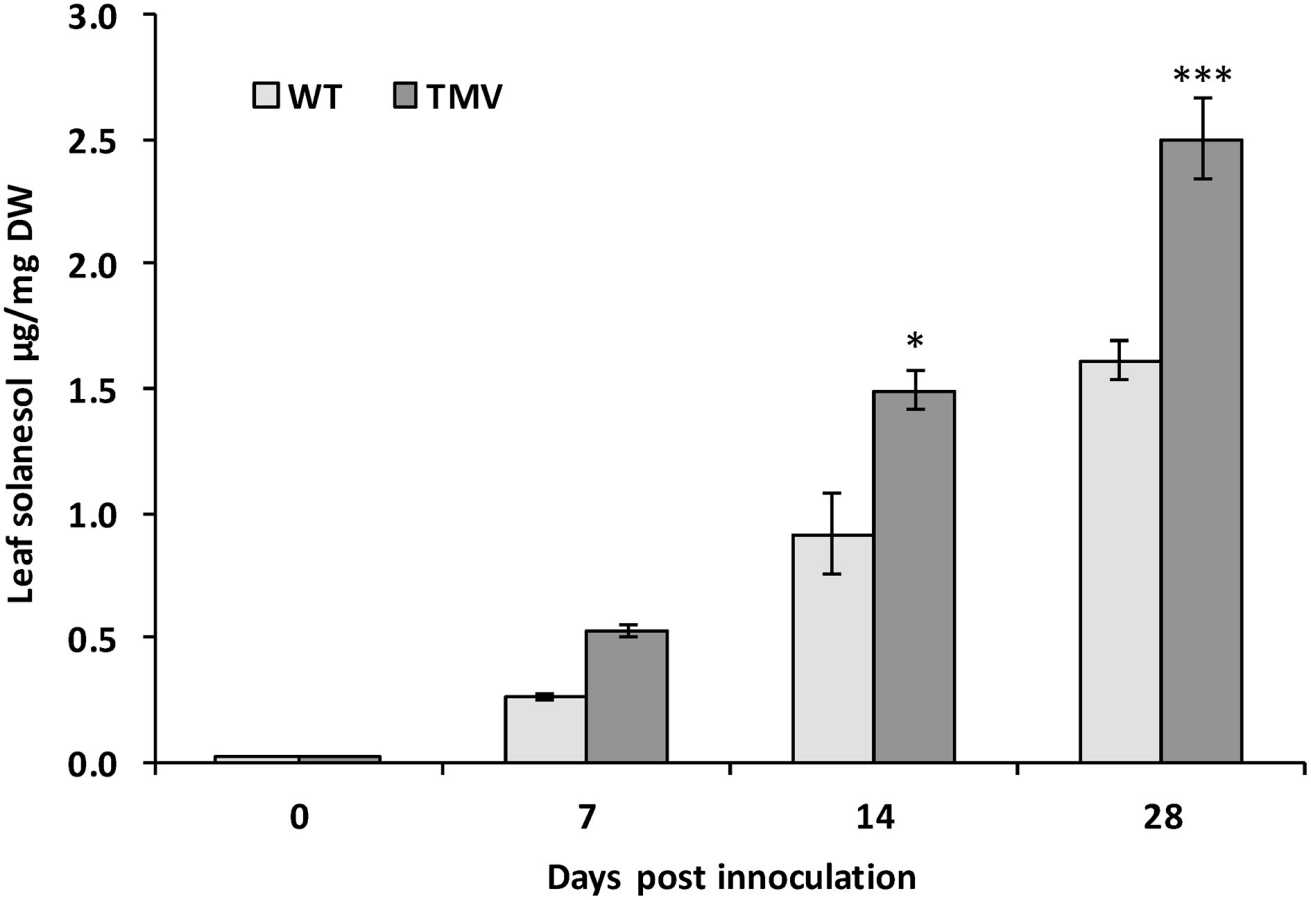
**Leaf Solanesol concentrations of *Solanum tuberosum* CV. Pentland Crown infected with TMV strain G3H15 measured over a period of 28 days**. Comparison of leaf solanesol concentrations (μg/g DW) between wild type and TMV infected *Solanum tuberosum* CV. Pentland Crown plants measured over a period of 28 days. Bars represent the mean value of three biological replicates. Statistical analysis was performed using Student's *t*-test and significance of differences are indicated (^*^P < 0.05, ^***^P < 0.001).

In tobacco mosaic virus (TMV) resistant tobacco there is a large (up to 8-fold) increase in leaf solanesol content when challenged with the TMV virus (Bajda et al., 2009). In the current study it was investigated whether a similar effect could be observed in potato. Consequently a mosaic virus resistant potato variety, *Solanum tuberosum* cv. Pentland Crown (Valkonen et al., 1994), was exposed to TMV strain G3H15 over a period of 28 days and the solanesol content measured. A 55% increase in solanesol content in leaves from the TMV infected plants was measured 28 days post infection (Figure 3).

### Effects of tuber greening on solanesol content

**Figure 4:**
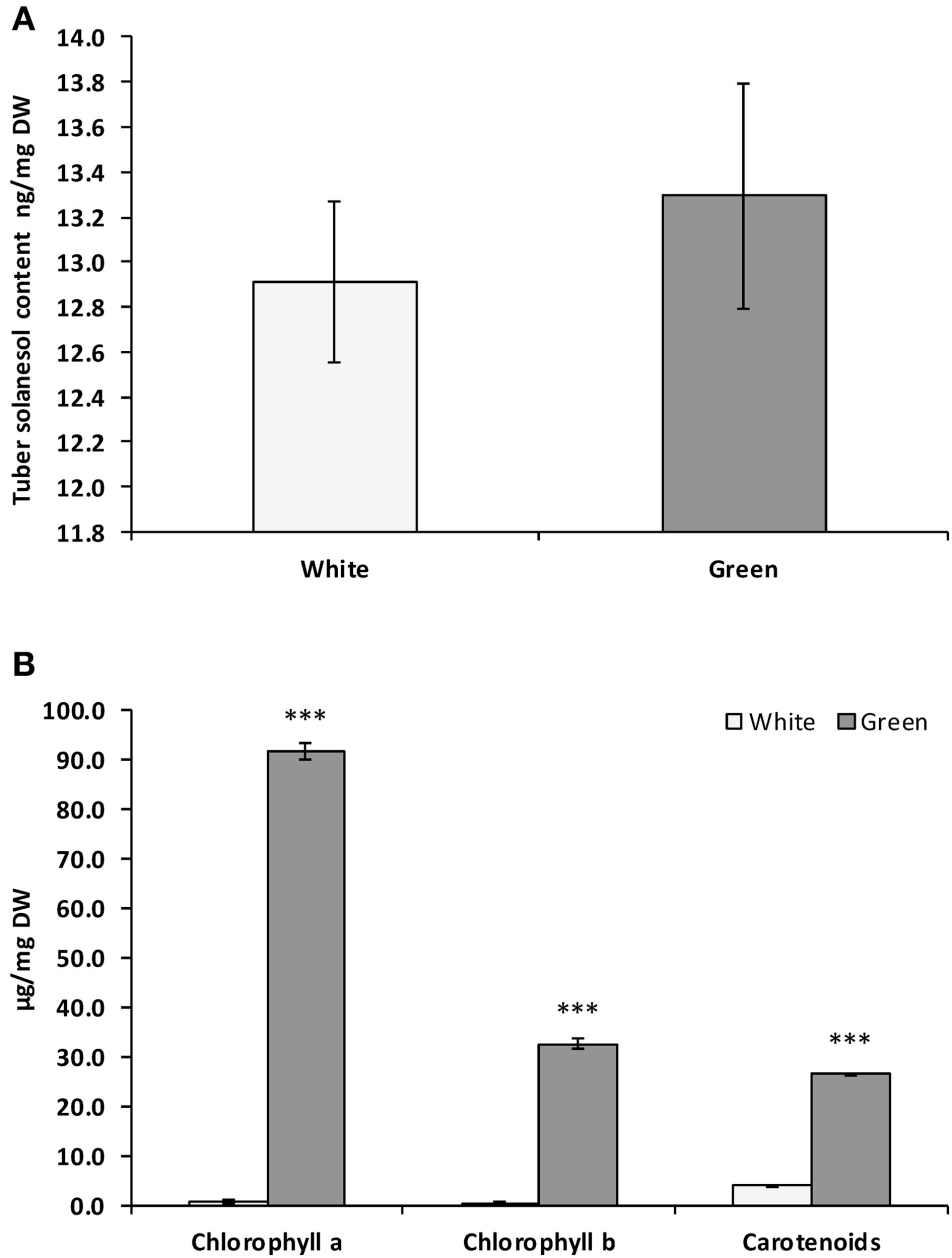
**Comparison of solanesol, chlorophylls and carotenoid content in *Solanum tuberosum* CV. Desiree white and green tubers**. Comparison of **(A)** solanesol (ng/mg DW), **(B)** chlorophyll a, chlorophyll b and carotenoid content (μg/mg DW) in dark stored (white) and light exposed (green) tubers cv. Desiree. Values shown are the mean of three replicates and the error bars shown are the mean of the standard error. Statistical analysis was performed using Student's *t*-test and significance of differences are indicated (^***^P < 0.001).

Solanesol accumulation has only been reported in green tissues (leaves and stems) and is thought to be synthesized in chloroplasts (Rodríguez-Concepción and Boronat, 2002). In potato (cv. Desiree), levels of solanesol measured in tubers was very low (Figure 4A), approximately 1000 fold lower than in leaves. On exposure to light, potato tuber amyloplasts differentiate to chloroplasts and chlorophyll can be observed to accumulate in tuber tissues (Zhu et al., 1984). We investigated whether the greening process in potato tubers was also associated with an increase in solanesol content. There was no significant difference in solanesol content in green tubers compared with non-green tubers, despite the increase in chlorophyll and carotenoid levels (Figures 4A,B).

### Transient expression of candidate genes

Transcriptomic analysis revealed that the expression levels of several MEP pathway genes increased on exposure to elevated temperature (Table 5A, Table S4), a condition where solanesol content increased ca. 6-fold. Furthermore inspection of the QTL for solanesol content revealed that a gene encoding solanesyl diphosphate synthase (SDS) mapped within the QTL interval for the solanesol leaf content QTL observed in year 1 on linkage group VII. In order to link the expression levels of these genes to solanesol content more closely, selected genes from the MEP pathway were expressed transiently in *N. benthamiana* and the effects on solanesol content were monitored.

**Figure 5:**
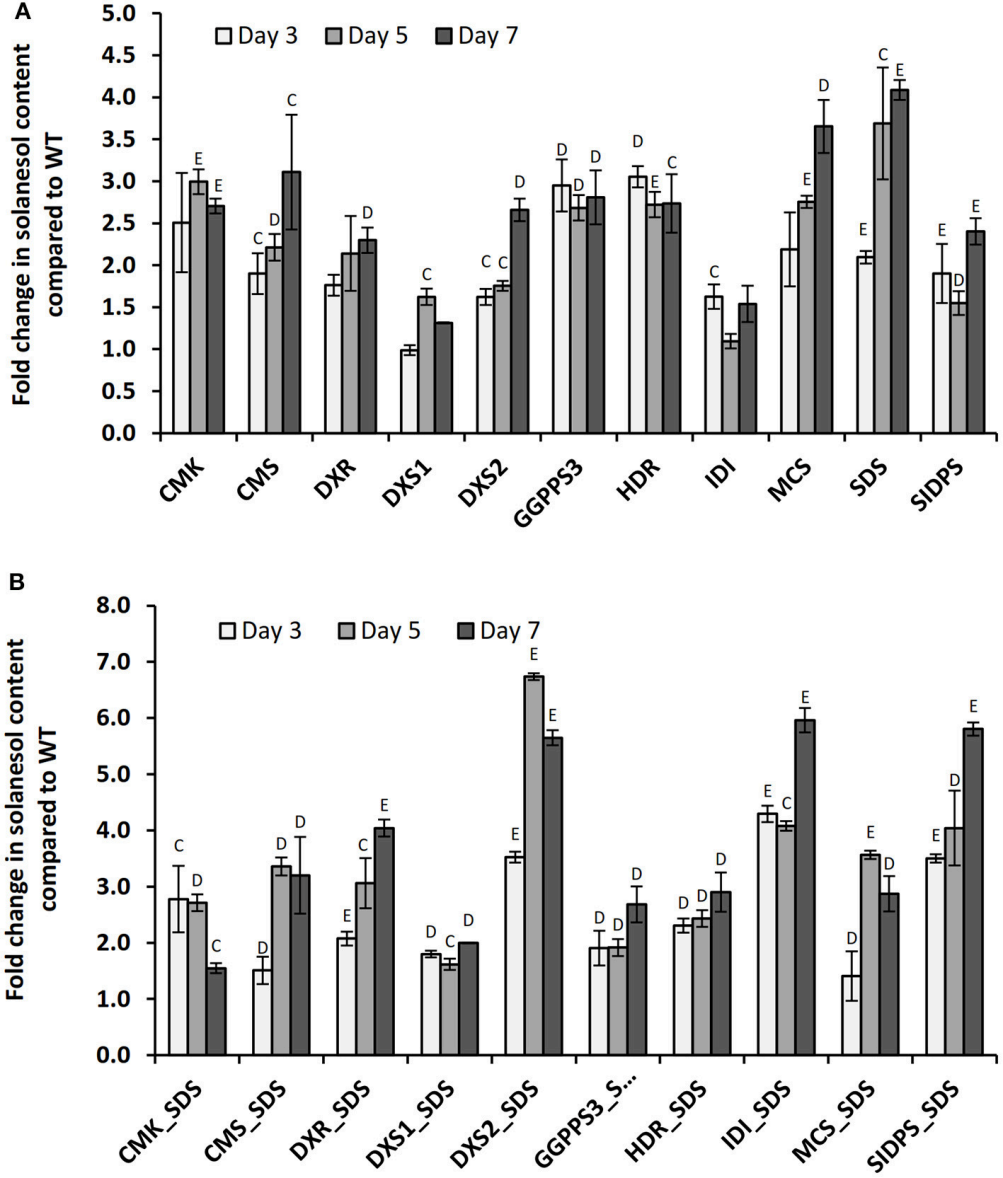
**Changes in leaf solanesol content in *N. benthamiana* plants transiently expressing MEP pathway genes**. Comparison of the relative fold changes in leaf solanesol content relative to the mock inoculated WT *N. benthamiana* plants in plants **(A)** transiently expressing single MEP pathway genes and **(B)** co-expression of MEP pathway gene and SDS. Values shown are presented at 3 time points and are the mean of three biological replicates. Statistical analysis was performed using Student's *t*-test and the significances are indicated (C) *P* < 0.05, (D) *P* < 0.01, (E) *P* < 0.001.

A second uncharacterized putative *SDS* gene, designated *SIDPS*, and located on chromosome 8 (pseudomolecule PGSC0003DMB000000518), was also tested. The coding sequence of this gene was elucidated from 12.5 kb of genomic sequence, contains 12 exons and is thought to contain a plastid targeting motif according to ChloroP 1.1 (Emanuelsson et al., 1999). Further information on this gene is presented in Figure S3. All genes tested were introduced by Agro-infiltration and expression was verified by qRT-PCR (Figure 5, Figure S4). A slight yellowing of the area around the inoculation sites was observed in leaves expressing the SDS construct (Figure S5). The effects of co-expression with the plastid associated *SDS* gene located on chromosome 7 were also investigated (Figure 5B).

In summary, transient over-expression of all MEP genes resulted in large increases in leaf solanesol content compared with the mock inoculated WT plants at 3, 5, and 7 days post infiltration with the exception of DXS1 at time point 3 and IDI (PGSC0003DMT400018755) at time point 5 where levels were found to be similar to the control plants. Expression of the *SDS* gene resulted in the largest increase in leaf solanesol content at both the day 5 and 7 time points, where the levels detected were 3.69 and 4.09-fold higher than found in the WT plants respectively. At the day 3 time point, expression of the potato *HDR* gene gave the highest solanesol levels measured. Interestingly, expression of the DXS1 and IDI constructs resulted in a comparatively lower fold increases in solanesol levels over the WT plants compared to the other 8 genes tested.

In an attempt to further increase the levels of solanesol the effects of gene stacking were investigated. Individual MEP genes were co-expressed with SDS which had shown the highest increases in leaf solanesol content when singularly expressed (Figure 5A). As previously observed, all gene combinations significantly increased solanesol content compared with the WT controls. Co-expression of gene combinations *DXS2:SDS, DXR:SDS, IDI:SDS*, and *SIDPS:SDS* all significantly improved the levels of solanesol accumulated compared to a single gene construct. For example, transient expression of the *IDI* gene at the day 7 time point resulted in a 1.54-fold increase in solanesol compared with a 5.96-fold increase when co-expressed with SDS. For the genes *HDR, CMK, MCS*, and *GGPPS* no significant improvement in solanesol content was observed when co-expressed with SDS (Figure 5B).

## Discussion

In a bi-parental potato population (06H1), there was wide variation in leaf solanesol content ranging from 0.03 to 0.77%. The wide variation in solanesol content in genotypes from this population enabled a QTL approach to understand the genetic architecture of the leaf solanesol content trait. For this study we have developed a targeted LC/MS method for the accurate quantification of solanesol and the values reported here are within the range previously reported for potato (Table 1). Several moderate effect QTL were identified that individually explained up to 13.3% of solanesol variation in the first stage 2014 samples and 9.3% of the variation at the first developmental stage in 2015. Whilst one of the QTL was observed in both seasons, some QTL only had a significant effect in one of the seasons. This included a QTL on chromosome 7. Underlying this QTL is a gene encoding SDS, an enzyme known to be involved in the solanesol biosynthetic pathway. We could not identify any known MEP pathway genes within any of the other QTL intervals. In order to gain greater understanding of the solanesol biosynthetic pathway, a number of MEP pathway genes, many of which were up-regulated in transcriptomic datasets in biotic and abiotic stressed potato plants, were transiently over-expressed in *N. benthamiana* and the effect on solanesol content measured. Over-expression of all MEP and solanesol pathway genes tested resulted in enhanced solanesol content compared with mock inoculated plants. In the single gene constructs the largest increases were observed when *SDS* was expressed, followed by *MCS*, and *CMS*. In testing gene combinations, co-expression of some MEP genes with *SDS* had additive effects, such as *DXS2*:*SDS, IDI*:*SDS*, and *SIDPS*:*SDS*, whereas others did not as in the case of *HDR*:*SDS, CMK*:*SDS*, and *DXS1*:*SDS*, possibly reflecting how manipulation of the solanesol biosynthetic pathway may give rise to new bottlenecks. Using this information informed decisions can be made for the effective stable metabolic engineering of the solanesol biosynthetic pathway.

Although, there was a genetic effect on solanesol content we also observed major differences in solanesol levels between the two growth seasons. The broad sense heritabilities for solanesol were 0.616 (2014, stage one), 0.598 (2014, stage two), 0.664 (2015, stage one), 0.864 (2015, stage two) suggesting a moderately high level of genetic control for the four traits but also reflecting a large non-genetic element in control of its level. The most notable difference in meteorological data between the two seasons was in average daily temperatures, with 2015 being cooler for the months of May, June and July. In general, for most genotypes, solanesol content was lower in the cooler 2015 season with a reduction in the median solanesol value of the population from 2.19 to 2.05 μg/mg and so we investigated the effects of temperature on leaf solanesol content in controlled environment experiments. The controlled environment experiments were carried out at higher temperatures than experienced in the field trials but still within the range of temperatures experienced in many major potato growing regions. Within seven days of exposure to moderately elevated temperature, leaf solansesol content increased dramatically (ca. 6-fold). Transcriptomic analysis of leaf samples from the two temperature regimes highlighted increases in expression levels of MEP and solanesol pathway genes, such as *SDS*, which was consistent with the effects on solanesol content on transient expression in *N. benthamiana*.

Previous studies have demonstrated significant increases (up to 7-fold) in the content of solanesol in infected leaves of resistant tobacco plants (*N. tabacum* NN) after TMV infection (Bajda et al., 2009). Application of exogenous hydrogen peroxide resulted in a two-fold increase in solanesol content potentially indicating a link with redox buffer status (Bajda et al., 2009). In the current study, we also observed an increase in solanesol content after TMV infection of resistant potato, however the increase was much less pronounced than reported for tobacco. We extend these observations by demonstrating a strong effect of elevated temperature on solanesol content. Interestingly, potato plants grown under identical elevated temperature conditions to those reported here were associated with a significantly greater degree of reduction in the leaf glutathione pool than at the lower temperature (Hancock et al., 2014) again indicating an association between redox buffer status and solanesol content. It has been suggested that polyisoprenoids such as solanesol function as modulators of the physical properties of biological membranes by impacting on membrane fluidity. Adjustment of membrane fluidity maintains an environment suitable for the function of critical integral membrane proteins during abiotic stress, e.g., photosynthetic machinery proteins in plants (Upchurch, 2008) or heat-shock proteins (HSP) in mammals (Vigh et al., 2007) and may explain the role of solanesol at elevated temperature. Future studies may investigate whether the genotypic variation in solanesol content observed in the 06H1 population is associated with responses to heat stress.

The solanesol yield per hectare, based on the maximum level observed in genotypes from the 06H1 population and a haulm dry matter content of 5.1 tons per hectare (Carruthers and Pirie, 1975) is estimated to be ca. 39 kg/hectare. Haulm destruction is an important step in potato agronomy, used to stop bulking and optimize the marketable tuber yield (SAC technical note T491)^1^. Haulm destruction can be brought about by chemical or mechanical means or a combination of bkoth. Following desiccation, potato haulm is not harvested and is generally plowed into the field. Previous studies have considered the potential of potato haulm as a source of fiber and protein (Carruthers and Pirie, 1975) demonstrating yields of extractable protein of up to 600 kg/ha. Despite these observations, potato haulm remains essentially a waste product of production. In this study we have identified both genetic and environmental factors that impact on potato leaf solanesol content, a metabolite of significant commercial interest. The identification of these factors could lead to strategies for producing elevated levels of leaf solanesol using conventional breeding or by metabolic engineering. The potential of potato haulm as a commercial source of solanesol could add to the argument for using a biorefinery approach to potato production.

## Authors contributions

MT, DS, and RC designed the research; RC, GB, and SF performed the experimental analyses. MT, RC wrote the article with inputs from SF, GB.

## Funding

This work was supported through the European Union Framework Program 7 DISCO grant 613153 (From discovery to products: A next generation pipeline for the sustainable generation of high-value plant products).

### Conflict of interest statement

The authors declare that the research was conducted in the absence of any commercial or financial relationships that could be construed as a potential conflict of interest.

## Footnotes

^1^Potato Haulm Destruction in Conventional and Organic Crops. Technical Note T491, SAC Edinburgh.
