# Supplementary material for: Environmental and Genetic Factors Associated with Solanesol Accumulation in Potato Leaves
Source: Front Plant Sci. 2016 Aug 25;7:1263. doi: 10.3389/fpls.2016.01263 (PMC4996988; doi:10.3389/fpls.2016.01263)
Supplement: Supplementary file 1 [file Table1.pdf]

Table S1. Primer sequences used for cloning MEP pathway genes.

Gene	Transcript ID		Primer sequences (5' to 3')
DXS1	PGSC0003DMT400058821	Forward	GCGCCGTCTCGCTCGAATGGCTTTGTGTGCTTATGCA
		Reverse	GCGCCGTCTCGCTCGAAGCTTATGTCATGACCTCTAGAGC
DXS2	PGSC0003DMT400041591	Forward	GCGCCGTCTCGCTCGAATGGCAGTTTCTTCAGGTCC
		Reverse	GCGCCGTCTCGCTCGAAGCCTATAGCATTTTGGAGATTTGATTTA
DXR	PGSC0003DMT400062965	Forward	GCGCCGTCTCGCTCGAATGGCCCTCAATTTGCTTTCT
		Reverse	GCGCCGTCTCGCTCGAAGCTCATACGAGAGCTGGACTCA
CMS	PGSC0003DMT400046906	Forward	GCGCCGTCTCGCTCGAATGTCTACTCTTCAATTGGGTATC
		Reverse	GCGCCGTCTCGCTCGAAGCCTAAGATTCTTCAATGTTCAATATTC
CMK	PGSC0003DMT400058461	Forward	GCGCCGTCTCGCTCGAATGGCTTCCTGTAATATTCTTTGT
		Reverse	GCGCCGTCTCGCTCGAAGCTCAATTTACAGGCTCTTCTGAG
HDR	PGSC0003DMT400064783	Forward	GCGCCGTCTCGCTCGAATGGCTATTCCTCTCCAATTC
		Reverse	GCGCCGTCTCGCTCGAAGCTTAGGCCAATTGTAAGGCTTC
MCS	PGSC0003DMT400031768	Forward	GCGCCGTCTCGCTCGAATGGCGTCTTCACTATTTTTTTCA
		Reverse	GCGCCGTCTCGCTCGAAGCCTATTTCTTCATAAGAAGAACAACAG
IDI	PGSC0003DMT400018755	Forward	GCGCCGTCTCGCTCGAATGAGAGGAATTGATGGGAAC
		Reverse	GCGCCGTCTCGCTCGAAGCTTAAGTCAATTTGTGGATGGTTTTC
GGPPS3	PGSC0003DMT400097473	Forward	GCGCCGTCTCGCTCGAATGAGTCTTTCAACAACAATTACTAC
		Reverse	GCGCCGTCTCGCTCGAAGCTTAATTCTCTCTGTAAGCAATATAATC
SDS	PGSC0003DMT400018242	Forward	GCGCCGTCTCGCTCGAGCCATGATGTCTGTGAGTTGCCAT
		Reverse	GCGCCGTCTCGCTCGAAGCCTATTCAATTCTCTCCAGATTATAC
SIDPS	--	Forward	ATGATATTTTCAAAGGGTTTATCTCAG
		Reverse	CTATTTTGTTCTTGTGATGACTCTG
