# Supplementary material for: Environmental and Genetic Factors Associated with Solanesol Accumulation in Potato Leaves
Source: Front Plant Sci. 2016 Aug 25;7:1263. doi: 10.3389/fpls.2016.01263 (PMC4996988; doi:10.3389/fpls.2016.01263)
Supplement: Supplementary file 2 [file Table2.pdf]

Table S2. Leaf solanesol content (μg/mg) DW in the 06H1 population.

Genotype ID	clone	2014 stg1 r1	2014 stg1 r2	2014 stg1 r3	2014 stg1 avg	2014 stg2 R1	2014 stg2 R2	2014 stg2 R3	2014 stg2 avg	2015 stg1 r1	2015 stg1 r2	2015 stg1 r3	2015 stg1 avg	2015 stg2 r1	2015 stg2 r2	2015 stg2 r3	2015 stg2 avg
06.H.1 a 1	1	1.853	1.740	0.785	1.460	2.29	2.53	2.53	2.45	0.937	0.661	0.616	0.74	5.55	4.75	5.30	5.20
06.H.1 a 2	2	0.280	0.174	0.253	0.236	2.88	1.25	1.99	2.04	0.143	0.370	0.100	0.20	2.39	2.86	2.67	2.64
06.H.1 a 4	4	*	*	*	*	*	*	*	*	*	*	*	*	*	*	*	*
06.H.1 a 7	7	1.035	0.316	1.693	1.014	2.89	2.97	4.66	3.50	1.356	1.920	2.576	1.95	6.21	9.38	7.47	7.69
06.H.1 a 9	9	0.672	0.384	0.815	0.624	2.96	2.26	2.51	2.58	0.612	0.749	0.563	0.64	3.06	2.83	2.58	2.83
06.H.1 a 10	10	0.465	0.822	0.258	0.515	2.90	3.58	2.51	3.00	0.703	0.726	0.504	0.64	3.68	3.42	4.71	3.94
06.H.1 a 12	12	0.299	0.224	0.404	0.309	1.25	1.47	1.39	1.37	0.285	0.261	0.429	0.32	1.72	2.00	1.25	1.66
06.H.1 a 13	13	0.273	0.171	0.293	0.246	2.64	2.47	1.57	2.22	0.357	0.812	0.680	0.62	4.64	3.42	2.90	3.65
06.H.1 a 14	14	0.548	0.801	0.608	0.653	2.05	2.90	2.37	2.44	1.117	1.243	0.712	1.02	6.56	4.85	5.27	5.56
06.H.1 a 15	15	0.507	0.715	0.580	0.601	1.34	1.85	1.99	1.73	1.134	0.591	1.633	1.12	5.52	4.36	5.17	5.02
06.H.1 a 21	21	0.209	0.416	0.362	0.329	0.99	1.17	1.60	1.26	0.582	0.626	0.359	0.52	2.22	3.26	2.09	2.52
06.H.1 a 22	22	0.262	0.572	0.563	0.466	0.86	3.53	3.06	2.48	1.255	0.946	1.065	1.09	4.12	4.18	3.05	3.78
06.H.1 a 24	24	0.280	0.183	0.327	0.263	2.24	1.44	1.77	1.81	0.866	1.048	0.798	0.90	2.17	1.62	1.36	1.72
06.H.1 a 26	26	0.151	0.224	0.194	0.189	0.36	1.30	2.56	1.40	0.223	0.260	0.328	0.27	1.76	1.37	1.09	1.41
06.H.1 a 27	27	0.153	0.136	0.250	0.180	0.97	1.26	1.35	1.19	0.154	0.268	0.223	0.21	0.48	0.86	0.75	0.69
06.H.1 a 28	28	0.247	0.095	0.199	0.181	1.41	1.82	3.40	2.21	0.885	0.724	0.401	0.67	0.53	0.50	1.05	0.70
06.H.1 a 29	29	0.968	2.083	2.718	1.923	5.29	5.45	4.23	4.99	0.411	0.636	0.162	0.40	0.92	1.65	0.99	1.19
06.H.1 a 32	32	2.400	0.787	2.061	1.749	2.80	2.79	3.13	2.91	0.592	0.722	0.442	0.59	2.21	2.37	1.74	2.11
06.H.1 a 41	41	0.389	0.369	0.520	0.426	4.91	4.92	2.66	4.16	0.369	0.398	0.533	0.43	4.60	2.67	2.74	3.33
06.H.1 a 49	49	0.560	0.787	0.426	0.591	5.50	4.35	4.90	4.92	0.378	0.295	0.152	0.28	1.02	1.64	1.15	1.27
06.H.1 a 50	50	0.681	0.693	0.689	0.688	3.58	1.99	1.10	2.22	0.453	0.788	0.955	0.73	0.62	0.63	0.88	0.71
06.H.1 a 51	51	0.569	0.950	1.071	0.863	4.59	3.31	4.87	4.26	0.371	0.268	0.262	0.30	4.18	4.82	4.86	4.62
06.H.1 a 57	57	0.262	0.274	0.309	0.282	0.62	1.08	1.26	0.99	0.539	1.607	1.749	1.30	4.64	3.26	3.91	3.94
06.H.1 a 58	58	0.077	0.078	0.090	0.082	2.12	2.97	2.07	2.38	0.019	0.152	0.901	0.36	2.73	3.85	3.59	3.39
06.H.1 a 61	61	0.357	0.361	0.262	0.327	2.61	3.04	2.50	2.72	0.692	0.483	0.484	0.55	1.51	1.25	1.85	1.53
06.H.1 a 64	64	0.806	0.801	0.599	0.736	2.75	1.82	4.67	3.08	0.595	0.864	1.187	0.88	1.89	1.49	1.45	1.61
06.H.1 a 65	65	2.462	0.609	0.781	1.284	2.74	2.03	2.99	2.59	1.575	0.864	1.949	1.46	2.74	3.13	2.48	2.78
06.H.1 a 66	66	1.700	1.272	1.448	1.473	1.85	1.78	2.39	2.01	1.390	1.217	1.692	1.43	2.19	2.26	3.90	2.78
06.H.1 a 69	69	0.333	0.455	0.441	0.410	2.05	1.09	1.72	1.62	0.258	0.477	0.491	0.41	2.03	1.46	1.54	1.67
06.H.1 a 71	71	*	*	*	*	*	*	*	*	0.196	0.279	0.608	0.36	1.24	0.69	0.85	0.93
06.H.1 a 76	76	0.187	0.231	0.110	0.176	1.88	0.86	1.11	1.28	0.252	0.271	0.748	0.42	1.17	1.06	0.86	1.03
06.H.1 a 78	78	0.389	0.702	0.111	0.401	1.64	3.39	2.47	2.50	0.507	0.502	0.193	0.40	2.55	3.31	2.79	2.88
06.H.1 a 79	79	0.502	0.339	0.434	0.425	3.48	3.42	1.38	2.76	0.582	0.553	0.631	0.59	0.77	0.75	0.78	0.77
06.H.1 a 80	80	0.112	0.250	0.184	0.182	0.84	1.34	1.84	1.34	0.587	0.598	0.479	0.55	2.95	4.47	3.51	3.64
06.H.1 a 81	81	0.227	0.289	0.209	0.242	2.45	2.55	2.03	2.35	0.323	0.780	0.528	0.54	0.85	1.28	1.62	1.25
06.H.1 a 83	83	0.243	0.868	0.165	0.425	1.08	2.99	1.49	1.85	0.130	0.316	0.357	0.27	0.60	1.28	0.97	0.95
06.H.1 a 86	86	0.147	0.221	0.213	0.194	1.97	1.34	1.61	1.64	0.273	0.237	0.233	0.25	0.56	0.58	0.43	0.53
06.H.1 a 87	87	0.890	0.904	0.802	0.865	2.30	2.24	2.23	2.25	0.754	0.714	0.254	0.57	1.11	2.98	2.90	2.33
06.H.1 a 88	88	0.357	0.450	0.436	0.414	2.32	2.28	5.05	3.22	0.691	1.025	0.551	0.76	2.61	3.43	3.93	3.33
06.H.1 a 89	89	0.516	1.445	0.872	0.944	2.44	2.17	2.57	2.39	0.449	1.180	0.114	0.58	0.84	1.15	0.99	0.99
06.H.1 a 90	90	0.678	1.548	1.065	1.097	0.94	2.13	3.48	2.18	1.989	0.339	0.763	1.03	2.40	1.93	1.61	1.98
06.H.1 a 95	95	0.106	0.278	0.120	0.168	2.52	2.25	1.96	2.24	0.776	0.643	0.622	0.68	0.86	0.79	2.22	1.29
06.H.1 a 97	97	1.082	0.640	0.275	0.666	2.14	4.53	3.03	3.24	0.589	0.679	1.016	0.76	1.74	1.78	1.19	1.57
06.H.1 a 99	99	0.306	0.220	0.271	0.266	1.66	2.75	1.16	1.86	0.964	0.253	0.138	0.45	0.58	1.12	1.08	0.93
06.H.1 a 100	100	0.383	0.594	0.433	0.470	1.69	1.68	2.82	2.07	0.780	1.038	1.050	0.96	1.13	1.25	1.83	1.41
06.H.1 a 107	107	0.310	0.241	0.108	0.220	3.53	3.03	1.53	2.70	0.534	0.550	0.873	0.65	2.79	3.47	2.08	2.78
06.H.1 a 108	108	0.143	0.151	0.465	0.253	1.87	1.91	1.86	1.88	0.286	0.998	0.286	0.52	3.97	3.35	5.37	4.23
06.H.1 a 110	110	0.256	0.202	0.300	0.253	1.59	2.53	4.02	2.71	0.481	0.659	0.629	0.59	0.95	0.71	0.66	0.77
06.H.1 a 112	112	0.415	1.298	0.738	0.817	2.11	1.98	1.60	1.89	0.215	0.832	1.181	0.74	4.02	4.90	3.82	4.24
06.H.1 a 113	113	0.231	0.366	0.237	0.278	2.99	2.67	2.75	2.80	*	*	*	*	*	*	*	*
06.H.1 a 116	116	0.229	0.261	0.310	0.267	1.73	1.12	1.66	1.50	0.487	0.415	0.460	0.45	3.63	3.37	4.15	3.72
06.H.1 a 121	121	2.019	0.499	0.624	1.047	4.15	3.51	2.11	3.26	0.199	0.719	0.452	0.46	3.33	5.56	2.69	3.86
06.H.1 a 122	122	*	*	*	*	*	*	*	*	*	*	*	*	*	*	*	*
06.H.1 a 123	123	0.251	0.367	0.323	0.314	2.03	2.02	3.11	2.390	0.383	0.651	1.086	0.71	2.82	2.79	2.49	2.70
06.H.1 a 126	126	0.192	0.252	0.518	0.321	3.07	2.57	3.35	3.00	1.480	2.073	1.676	1.74	2.10	2.58	1.58	2.09
06.H.1 a 127	127	0.521	0.151	0.088	0.253	2.03	1.27	1.84	1.71	0.406	0.572	0.835	0.60	3.29	2.64	3.17	3.03
06.H.1 a 128	128	0.613	0.158	0.431	0.401	2.82	2.97	2.66	2.82	0.555	0.358	0.780	0.56	3.69	4.12	3.27	3.69
06.H.1 a 129	129	0.726	0.660	0.749	0.712	2.91	3.08	2.79	2.93	2.864	2.411	2.436	2.57	3.03	3.16	2.28	2.82
06.H.1 a 130	130	0.427	1.321	0.363	0.704	2.16	1.81	1.46	1.81	1.081	0.836	0.962	0.96	3.99	4.43	3.41	3.94
06.H.1 a 135	135	0.476	0.698	0.863	0.679	3.27	2.90	2.37	2.85	1.065	1.240	0.951	1.09	1.41	1.41	1.31	1.38
06.H.1 a 136	136	0.206	0.272	0.273	0.251	1.53	2.75	2.19	2.16	0.405	0.262	0.081	0.25	1.92	1.60	2.66	2.06
06.H.1 a 137	137	0.523	0.302	0.429	0.418	0.91	0.91	1.02	0.95	0.900	0.625	0.639	0.72	1.56	2.91	1.60	2.02
06.H.1 a 138	138	0.282	0.535	1.491	0.769	2.61	2.51	2.56	2.56	0.656	0.556	0.141	0.45	4.80	3.83	3.56	4.06
06.H.1 a 140	140	0.519	0.362	0.545	0.475	3.20	3.95	1.62	2.93	1.698	1.411	2.745	1.95	2.29	2.44	2.80	2.51
06.H.1 a 141	141	0.960	0.932	0.836	0.910	3.34	2.88	3.66	3.29	0.204	0.601	0.584	0.46	0.60	0.43	0.21	0.41
06.H.1 a 143	143	0.226	0.235	0.280	0.247	0.66	0.72	0.71	0.690	1.195	0.565	0.203	0.65	1.30	0.78	1.16	1.08
06.H.1 a 144	144	0.208	0.188	0.498	0.298	2.61	3.04	2.01	2.56	1.111	0.977	0.707	0.93	3.22	2.13	2.32	2.56
06.H.1 a 145	145	0.638	0.232	0.384	0.418	2.76	2.60	1.12	2.16	0.488	0.268	0.484	0.41	1.36	1.79	1.44	1.53
06.H.1 a 147	147	0.296	0.327	0.366	0.330	2.94	4.26	3.40	3.530	1.380	1.245	0.970	1.20	2.15	1.96	1.79	1.96
06.H.1 a 148	148	0.216	0.455	0.849	0.507	0.81	2.06	1.66	1.51	3.256	2.305	2.088	2.55	4.24	3.69	4.21	4.05
06.H.1 a 149	149	*	*	*	*	*	*	*	*	*	*	*	*	*	*	*	*
06.H.1 a 151	151	1.049	0.214	0.439	0.567	1.25	1.91	1.17	1.44	0.335	0.365	0.305	0.34	3.72	2.72	2.84	3.09
06.H.1 a 152	152	0.299	0.230	0.919	0.482	1.44	2.94	2.83	2.40	0.323	0.132	0.202	0.22	1.93	1.31	1.29	1.51
06.H.1 a 153	153	0.483	0.312	0.416	0.403	2.65	2.57	2.27	2.50	0.588	1.273	0.968	0.94	2.73	1.31	1.31	1.78
06.H.1 a 154	154	0.322	0.129	0.138	0.196	4.36	2.24	2.47	3.02	0.519	0.437	0.590	0.52	3.34	3.67	4.34	3.78
06.H.1 a 155	155	0.294	0.259	0.418	0.323	3.04	1.32	4.59	2.98	0.311	0.342	0.964	0.54	1.22	2.14	1.35	1.57
06.H.1 a 156	156	0.161	0.138	0.284	0.195	1.96	2.30	2.25	2.17	0.726	0.353	0.468	0.52	3.03	3.00	2.44	2.83
06.H.1 a 158	158	0.670	0.409	0.193	0.424	2.13	2.41	1.93	2.16	0.349	0.461	0.101	0.30	3.37	2.80	2.78	2.98
06.H.1 a 161	161	*	*	*	*	*	*	*	*	*	*	*	*	*	*	*	*
06.H.1 a 164	164	*	*	*	*	*	*	*	*	*	*	*	*	*	*	*	*
06.H.1 a 165	165	0.179	0.426	0.349	0.318	2.22	2.00	1.82	2.01	0.440	0.291	0.431	0.39	3.08	4.56	4.07	3.90
06.H.1 a 166	166	0.757	1.152	1.392	1.100	1.25	1.44	1.53	1.41	0.143	1.563	0.309	0.67	2.61	1.71	2.26	2.19
06.H.1 a 168	168	0.890	0.558	0.638	0.695	2.36	2.46	1.66	2.16	0.072	0.653	1.318	0.68	4.03	3.10	3.71	3.62
06.H.1 a 169	169	0.594	0.401	0.266	0.421	2.06	2.31	3.01	2.46	0.321	0.186	0.395	0.30	1.94	1.87	2.45	2.09
06.H.1 a 170	170	0.361	0.470	0.468	0.433	2.15	4.36	4.09	3.53	0.687	0.157	0.594	0.48	1.57	1.10	1.01	1.23
06.H.1 a 171	171	1.270	0.674	0.832	0.925	3.77	4.42	2.91	3.70	0.248	1.884	0.542	0.89	4.05	3.79	3.95	3.93
06.H.1 a 172	172	0.557	0.248	0.605	0.470	2.85	3.15	3.38	3.13	0.217	0.420	0.287	0.31	2.35	3.11	3.34	2.93
06.H.1 a 173	173	0.163	0.660	0.496	0.440	2.49	4.25	3.12	3.29	0.693	0.516	0.529	0.58	2.06	2.21	1.88	2.05
06.H.1 a 174	174	0.906	0.635	0.757	0.766	3.28	2.64	3.19	3.04	0.546	0.423	0.182	0.38	3.35	1.79	2.06	2.40
06.H.1 a 177	177	0.603	1.039	0.893	0.845	1.29	1.27	1.27	1.28	0.457	0.690	0.787	0.64	1.16	2.27	1.75	1.73
06.H.1 a 179	179	0.415	0.479	0.390	0.428	2.37	2.87	3.27	2.84	0.364	1.568	0.363	0.76	2.03	1.78	1.71	1.84
06.H.1 a 180	180	0.306	0.532	0.531	0.456	3.06	2.96	2.03	2.68	0.740	0.985	0.299	0.67	1.46	1.42	1.19	1.35
06.H.1 a 181	181	0.649	0.437	0.598	0.561	0.89	1.10	0.58	0.86	0.352	1.081	0.397	0.61	1.60	1.62	1.09	1.44
06.H.1 a 183	183	0.441	0.606	0.321	0.456	3.27	3.15	1.65	2.69	0.163	0.152	0.175	0.16	3.46	5.71	4.10	4.42
06.H.1 a 184	184	0.679	0.539	0.380	0.533	0.58	0.69	1.03	0.77	0.552	0.854	0.821	0.74	2.42	1.25	1.79	1.82
06.H.1 a 185	185	0.800	1.238	0.649	0.896	1.52	2.35	1.65	1.84	0.155	0.238	0.392	0.26	4.68	5.17	7.23	5.69
06.H.1 a 187	187	0.119	0.251	0.354	0.241	1.41	0.98	0.84	1.07	0.215	0.369	0.479	0.35	1.95	2.28	1.38	1.87
06.H.1 a 188	188	0.085	0.455	0.457	0.333	1.57	2.66	3.18	2.47	1.187	2.151	1.250	1.53	3.63	2.00	2.29	2.64
06.H.1 a 189	189	0.432	0.174	0.243	0.283	0.31	0.28	0.37	0.32	0.351	0.319	0.465	0.38	2.19	2.58	2.21	2.33
06.H.1 a 192	192	0.361	0.201	0.192	0.251	4.38	4.10	3.57	4.02	0.151	0.942	0.150	0.41	2.82	4.33	2.82	3.32
06.H.1 a 193	193	0.381	0.589	0.251	0.407	0.99	1.18	1.33	1.16	1.172	0.647	1.086	0.97	5.84	7.97	7.55	7.12
06.H.1 a 199	199	0.107	0.155	0.428	0.230	0.86	1.15	1.60	1.20	1.029	0.191	1.000	0.74	1.35	2.10	2.31	1.92
06.H.1 a 200	200	0.899	0.404	0.526	0.610	2.91	2.33	2.43	2.56	0.547	1.257	0.948	0.92	3.84	4.91	3.84	4.20
06.H.1 a 202	202	1.013	1.469	1.360	1.280	3.94	3.95	4.13	4.01	1.113	0.530	1.004	0.88	3.42	3.89	4.67	3.99
06.H.1 a 204	204	1.200	0.716	0.787	0.901	4.34	4.54	4.38	4.42	0.823	1.366	1.091	1.09	1.07	1.18	1.37	1.20
06.H.1 a 205	205	0.215	0.236	0.133	0.195	3.00	1.90	1.52	2.14	0.450	0.554	0.758	0.59	1.36	2.27	1.80	1.81
06.H.1 a 206	206	0.405	0.771	0.315	0.497	1.50	1.69	4.11	2.43	1.033	0.839	0.983	0.95	2.01	2.17	1.67	1.95
06.H.1 a 207	207	0.242	0.233	0.280	0.252	1.74	1.82	1.08	1.55	0.266	0.105	0.152	0.17	1.53	2.22	1.40	1.72
06.H.1 a 209	209	0.047	0.168	0.191	0.135	2.81	2.11	2.36	2.43	0.601	0.187	1.119	0.64	2.90	2.57	2.49	2.65
06.H.1 a 210	210	0.225	0.203	0.259	0.229	0.82	1.37	1.69	1.29	0.436	0.299	0.458	0.40	2.59	2.03	2.32	2.31
06.H.1 a 211	211	0.553	0.722	0.592	0.622	2.78	2.07	3.44	2.76	0.395	0.499	0.363	0.42	4.45	5.02	4.03	4.50
06.H.1 a 213	213	*	*	*	*	*	*	*	*	*	*	*	*	*	*	*	*
06.H.1 a 214	214	0.835	0.610	0.987	0.811	2.80	3.34	1.21	2.45	0.076	0.162	0.066	0.10	3.17	2.74	2.37	2.76
06.H.1 a 215	215	1.689	1.174	0.827	1.230	3.88	4.05	3.53	3.82	0.215	0.358	0.205	0.26	1.10	1.07	1.33	1.17
06.H.1 a 216	216	0.248	0.540	0.182	0.323	2.77	3.21	2.47	2.82	0.420	0.152	0.205	0.26	2.57	2.98	2.73	2.76
06.H.1 a 218	218	0.276	0.695	0.622	0.531	3.35	1.55	3.27	2.72	0.038	0.537	0.588	0.39	3.66	2.48	3.81	3.31
06.H.1 a 220	220	0.219	0.160	0.188	0.189	0.45	2.16	1.00	1.20	0.335	0.067	0.251	0.22	1.91	1.10	0.74	1.25
06.H.1 a 221	221	0.225	0.211	0.305	0.247	0.24	0.89	1.26	0.80	0.140	0.169	0.131	0.15	2.08	1.65	1.65	1.79
06.H.1 a 222	222	0.252	0.151	0.172	0.192	0.37	0.13	0.39	0.30	0.649	0.162	0.373	0.39	0.94	0.83	0.64	0.80
06.H.1 a 223	223	0.865	0.620	0.680	0.722	0.89	0.82	1.29	1.00	0.129	0.283	0.282	0.23	1.01	0.92	0.88	0.94
06.H.1 a 224	224	0.184	0.062	0.144	0.130	2.59	2.00	2.62	2.40	0.247	0.269	0.306	0.27	1.19	1.09	1.08	1.12
06.H.1 a 225	225	0.428	0.471	0.739	0.546	2.93	1.90	0.14	1.65	0.837	0.246	0.631	0.57	1.31	1.13	1.07	1.17
06.H.1 a 227	227	0.474	0.293	0.316	0.361	2.51	2.92	3.18	2.87	0.227	0.319	0.003	0.18	1.60	2.46	1.84	1.96
