# Supplementary material for: Environmental and Genetic Factors Associated with Solanesol Accumulation in Potato Leaves
Source: Front Plant Sci. 2016 Aug 25;7:1263. doi: 10.3389/fpls.2016.01263 (PMC4996988; doi:10.3389/fpls.2016.01263)
Supplement: Supplementary file 5 [file Image1.PDF]

Figure S1. Solanesol recovery rates from spiked extracts.

(a) Standard curve.

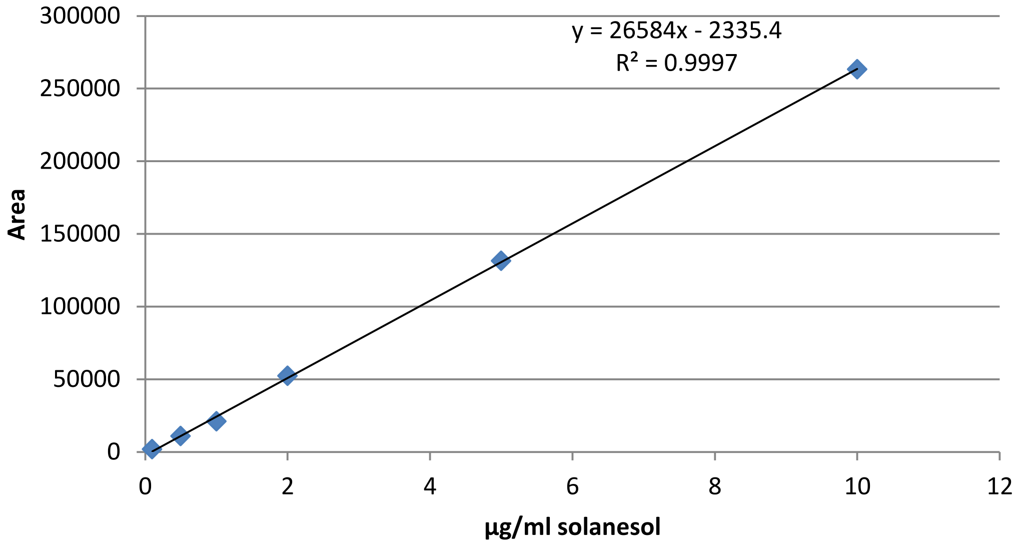

(b) Extracted standard.

Solanesol spike	Extract rea	% recovery
0.1	1652	90.6%
0.5	9963	91.6%
1	20087	95.8%
2	47123	90.2%
5	121092	92.1%
10	252828	96.1%

Solanesol s	Area mean
0.1	1823
0.5	10872
1	20968
2	52243
5	131421
10	263118
