# Supplementary material for: Environmental and Genetic Factors Associated with Solanesol Accumulation in Potato Leaves
Source: Front Plant Sci. 2016 Aug 25;7:1263. doi: 10.3389/fpls.2016.01263 (PMC4996988; doi:10.3389/fpls.2016.01263)
Supplement: Supplementary file 6 [file Image2.PDF]

Figure S2. Vector maps of 1α1 series Goldenbraid MEP constructs used in this study. MEP genes present are, (A) CMK, (B) CMS, (C) DXR, (D) DXS1, (E) DXS2, (F) GGPPS3, (G) HDR, (H) IDI, (I) MCS, (J) SDS, (K) SlDPS.

(A)

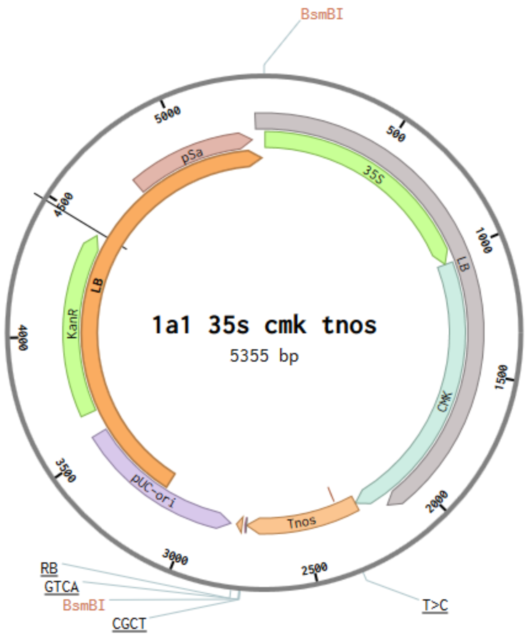

(B)

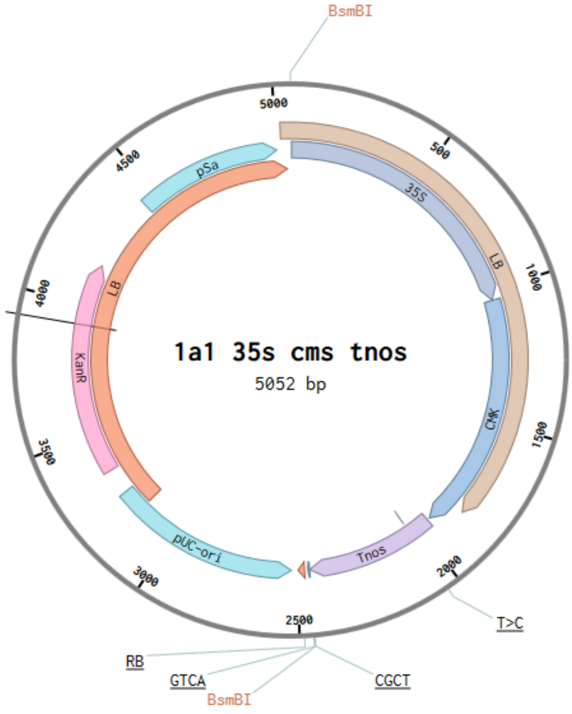

(C)

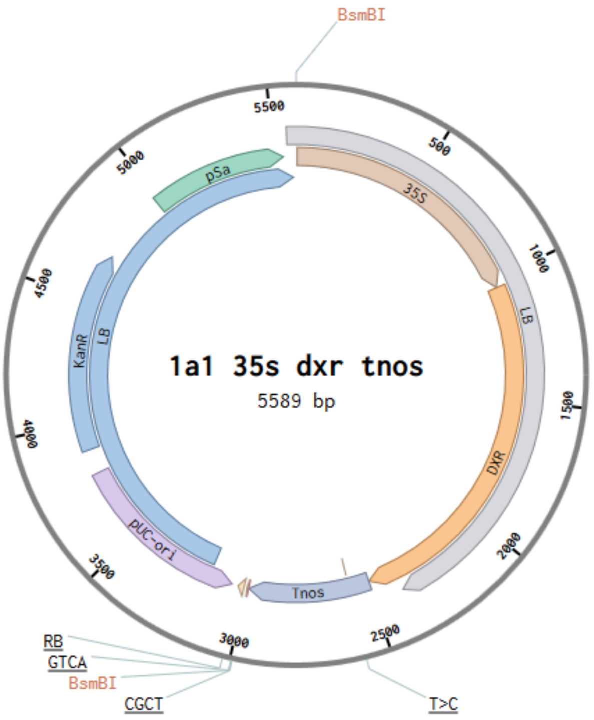
BsmBI
5500
500
5000
1000
4500
1500
4000
2000
3500
2500
3000
pSa
35S
LB
KanR
LB
DXR
pUC-ori
Tnos
1a1 35s dxr tnos
5589 bp
RB
GTCA
BsmBI
CGCT
T>C

(D)

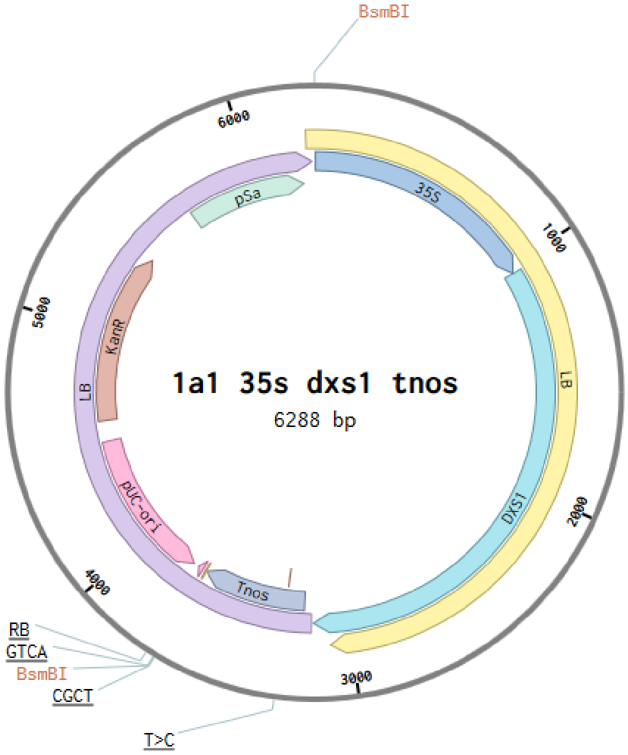
BsmBI
6000
1000
5000
2000
4000
3000
pSa
35S
KanR
LB
LB
DXS1
pUC-ori
Tnos
1a1 35s dxs1 tnos
6288 bp
RB
GTCA
BsmBI
CGCT
T>C

(E)

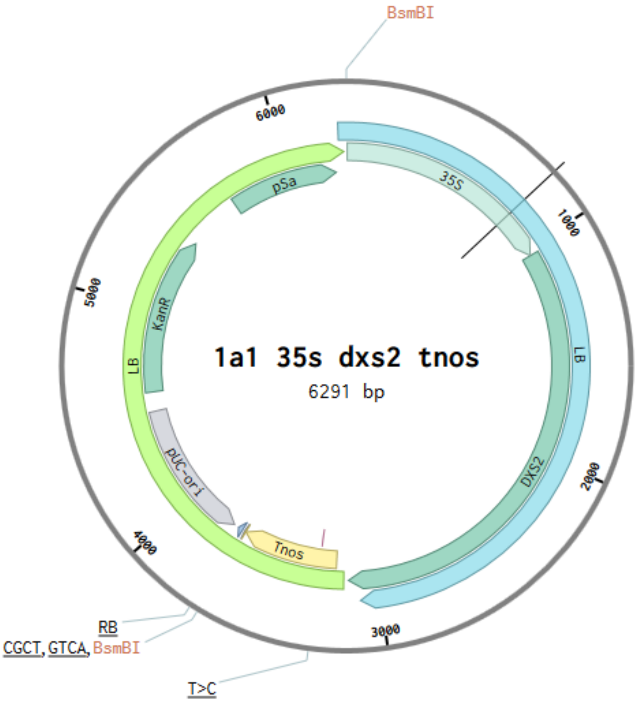
BsmBI
6000
35S
1000
5000
pSa
KanR
LB
1a1 35s dxs2 tnos
6291 bp
LB
DXS2
2000
pUC-ori
4000
Tnos
3000
RB
CGCT, GTCA, BsmBI
T>C

(F)

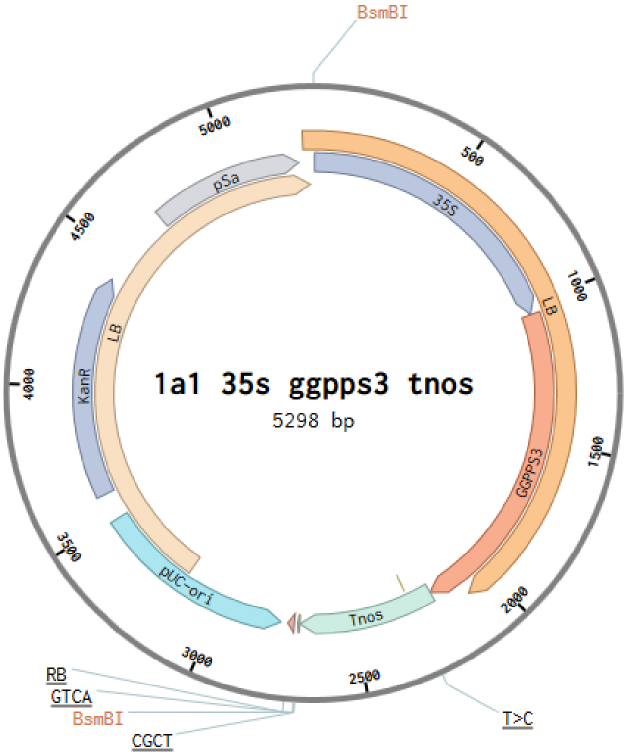
BsmBI
5000
500
4500
pSa
35S
1000
LB
LB
KanR
1a1 35s ggpps3 tnos
5298 bp
4000
GGPPS3
1500
3500
pUC-ori
Tnos
2000
3000
2500
RB
GTCA
BsmBI
CGCT
T>C

(G)

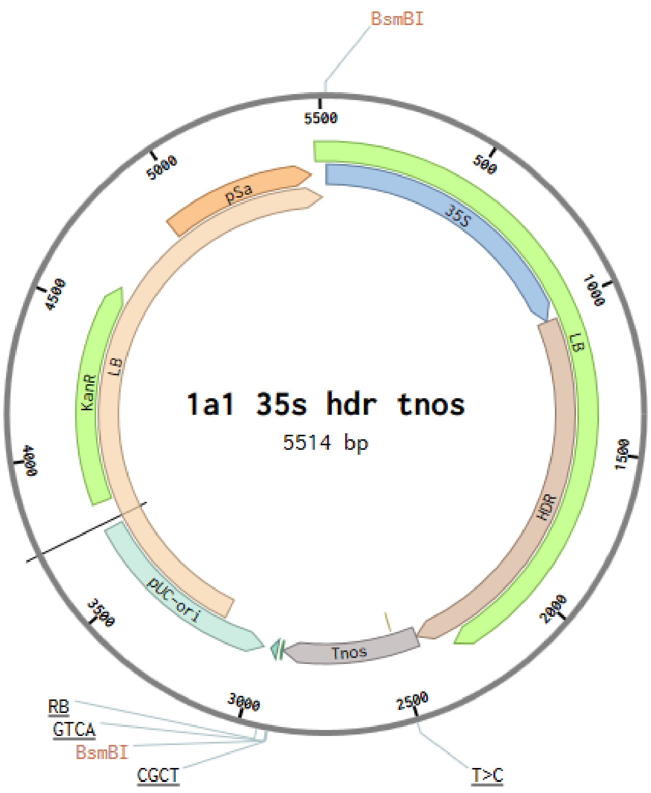
BsmBI
5500
500
5000
pSa
35S
1000
4500
LB
KanR
LB
1a1 35s hdr tnos
5514 bp
4000
1500
HDR
3500
pUC-ori
2000
Tnos
3000
2500
RB
GTCA
BsmBI
CGCT
T>C

(H)

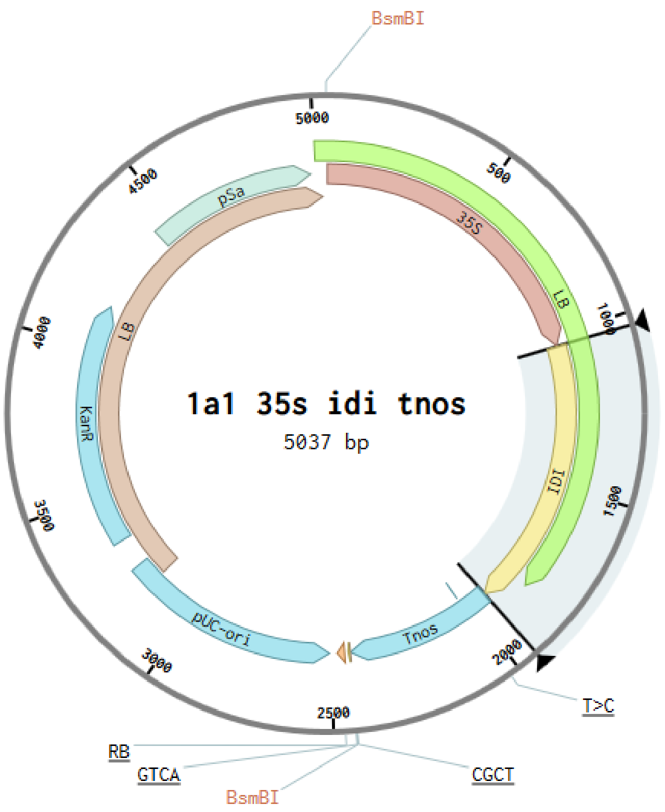
BsmBI
5000
500
4500
pSa
35S
LB
1000
4000
LB
KanR
1a1 35s idi tnos
5037 bp
IDI
1500
3500
pUC-ori
Tnos
3000
2000
T>C
2500
RB
GTCA
BsmBI
CGCT

(I)

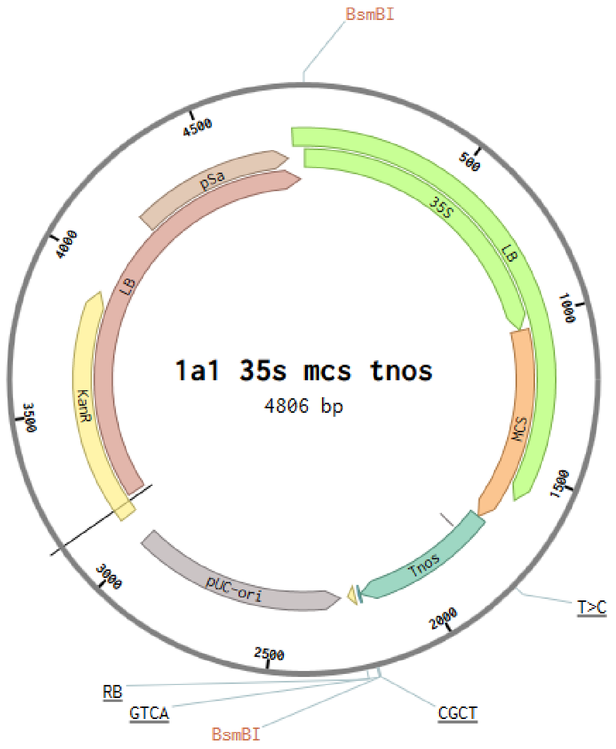
BsmBI
1a1 35s mcs tnos
4806 bp
35S
LB
MCS
Tnos
pUC-ori
KanR
LB
pSa
500
1000
1500
2000
2500
3000
3500
4000
4500
T>C
CGCT
RB
GTCA
BsmBI

(J)

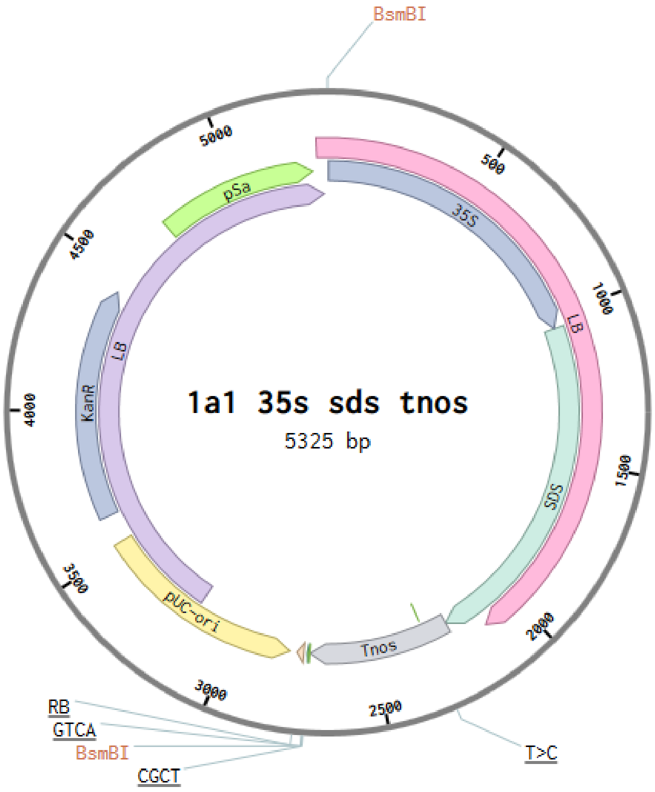
BsmBI
1a1 35s sds tnos
5325 bp
35S
LB
SDS
Tnos
pUC-ori
KanR
LB
pSa
500
1000
1500
2000
2500
3000
3500
4000
4500
5000
T>C
RB
GTCA
BsmBI
CGCT

(K)

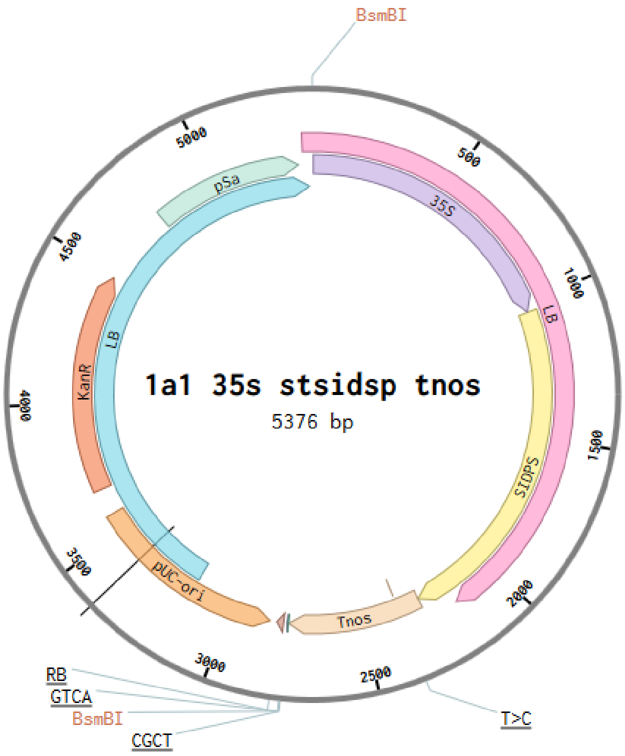

BsmBI
1a1 35s stsidsp tnos
5376 bp
35S
LB
SIDPS
Tnos
pUC-ori
KanR
LB
pSa
500
1000
1500
2000
2500
3000
3500
4000
4500
5000
RB
GTCA
BsmBI
CGCT
T>C
