# Supplementary material for: Environmental and Genetic Factors Associated with Solanesol Accumulation in Potato Leaves
Source: Front Plant Sci. 2016 Aug 25;7:1263. doi: 10.3389/fpls.2016.01263 (PMC4996988; doi:10.3389/fpls.2016.01263)
Supplement: Supplementary file 7 [file Image3.PDF]

Figure S3. Graphical representation of SlDSP exon intron structure and coding and protein sequence.

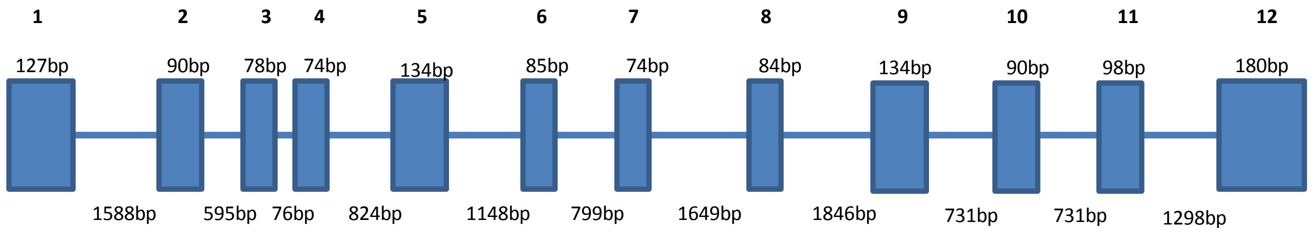

Coding sequence

>StSIDPS
ATGATATTTTCAAAGGGTTTATCTCAGATTTCCAGAAACCGCTTCAGTAGATGCCGATGGTTATTTTCAT
CGCGTCCCATCCAACAATTACATCAATCCAATCACATCCACGATCCTCCAAAGGTGATGGGTTGCAGAGT
AATTCATTCATGGGTTTCTAATGCTCTTAGTGGTATTGGGCAACAAATTCATCAGCAAAGCACTGCTGTA
GCAGAGGAGCAAGTGGACCCATTTTCCCTTGTTGCGGATGAATTATCCCTTCTGACAAACAGGCTGAGAT
CAATGGTAGTTGCTGAGGTCCCAAAGCTGGCTTCAGCTGCTGAATATTTCTTCAAACTGGGAGTTGAAGG
AAAGAGGTTTCGACCCACAGTTTTGCTGTTGATGGCAACTGCATTGAACGTACAGATTCCTAGATCTGCT
CCCCAGGTGGATGTTGATTCCTTGTCCGGGGATTTGCGTACAAGGCAGCAGTGTATAGCTGAGATCACTG
AGATGATCCATGTTGCCAGCCTACTTCATGATGATGTACTGGATGATGCTGACACAAGACGTGGGATAGG
TTCTTTAAACTTTGTGATGGGAAATAAGCTAGCTGTACTAGCCGGAGACTTTTTGCTTTCCCGAGCATGT
GTGGCACTTGCCTCCTTGAAGAACACAGAGGTTGTATCTCTTCTGGCAACTGTTGTGGAACATCTTGTTA
CTGGAGAGACAATGCAAATGACAACTTCTTCTGATGAACGTTGTAGCATGGAGTATTATATGCAGAAAAC
ATATTACAAGACTGCATCATTGATTTCAAACAGTTGCAAAGCGATTGCACTACTTGCTGGGCATACTGCT
GAAGTCTCCGTGCTGGCTTTTGACTACGGAAAAAATCTGGGATTGGCATTTCAATTAATAGATGATGTTC
TTGATTTCACGGGCACATCTGCAACCCTTGGCAAGGGTTCATTGTCTGATATTCGTCATGGGATTGTAAC
TGCCCCAATATTGTATGCCATGGAGGAATTTCCTCAACTGCGTACGCTGGTGGACCGAGGTTTTGATGAT
CCTGTAAATGTGGAGATCGCTCTAGACTACCTTGGGAAAAGCAGAGGGATACAGAGAACAAGAGAACTTG
CCAGAAAGCATGCTAGCCTTGCGTCAGCGGCAATTGACTCTCTTCCAGAAAGCGATGACGAGGAAGTTCA
GAGATCAAGAAGGGCACTTGTAGAACTTACTCACAGAGTCATCACAAGAACAAAATAG

Protein sequence

MIFSKGLSQISRNRFSRCRWLFSSRPIQQLHQSNHIHDPPKVMGCRVIHSWVSNALSGIGQQIHQQSTAV
AEEQVDPFSLVADELSLLTNRLRSMVVAEVPKLASAAEYFFKLGVEGKRFRPTVLLLMATALNVQIPRSA
PQVDVDSLSGDLRTRQQCIAEITEMIHVASLLHDDVLDDADTRRGIGSLNFVMGNKLAVLAGDFLLSRAC
VALASLKNTEVVSLLATVVEHLVTGETMQMTTSSDERCSMEYYMQKTYYKTASLISNSCKAIALLAGHTA
EVSVLAFDYGKNLGLAFQLIDDVLDFTGTSATLGKGSLSDIRHGIVTAPILYAMEEFPQLRTLVDRGFDD
PVNVEIALDYLGKSRGIQRTRELARKHASLASAAIDSLPESDDEEVQRSRRALVELTHRVITRTK
