# Supplementary material for: Environmental and Genetic Factors Associated with Solanesol Accumulation in Potato Leaves
Source: Front Plant Sci. 2016 Aug 25;7:1263. doi: 10.3389/fpls.2016.01263 (PMC4996988; doi:10.3389/fpls.2016.01263)
Supplement: Supplementary file 8 [file Image4.PDF]

Figure S4. Relative expression of transiently expressed potato MEP pathway genes in *N.benthamiana* determined by Universal Probe Library RT-PCR. Values shown are normalised to the day 3 gene timepoint. Error bars shown are the standard error of the mean. Primer and probe numbers of each assay are indicated in the table.

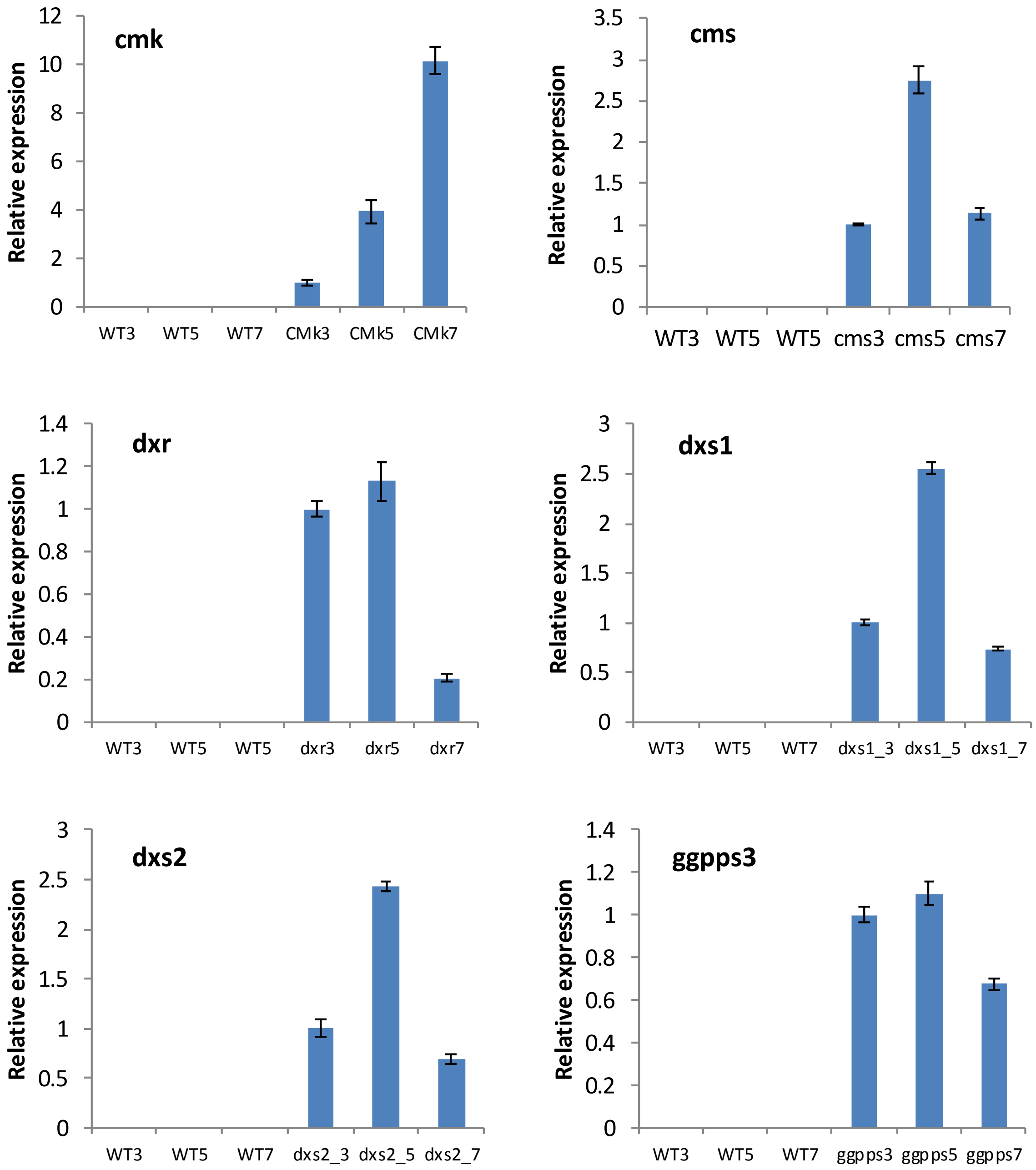

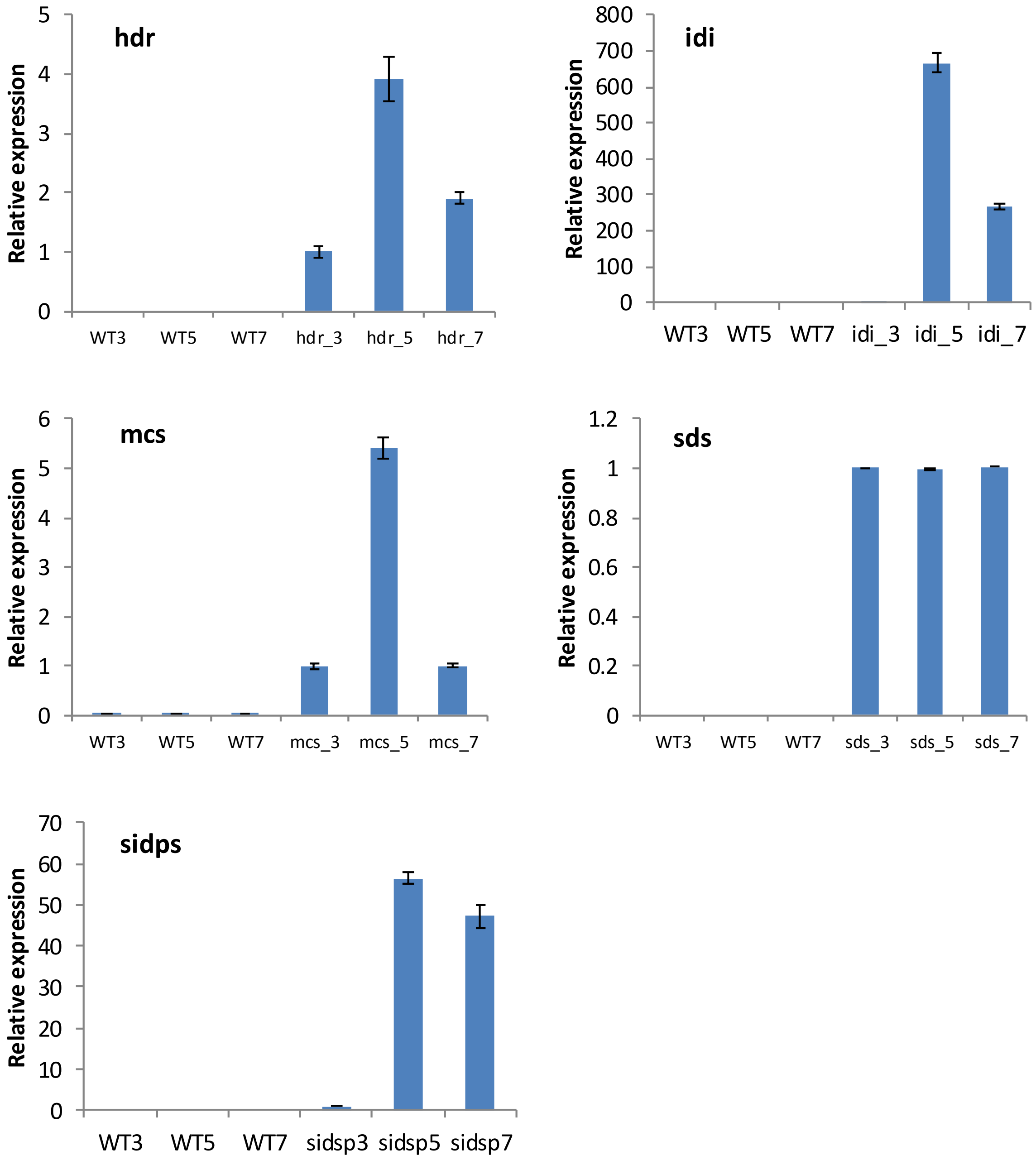

Gene	Forward primer	Reverse primer	Probe number
CMK	ggtctggtgagattggttctg	acccgtacagtaggctgctc	143
CMS	aaaggtcctgaaggatggttg	gcctctttgattgtggcttt	132
DXR	ccatccccaatccatcatac	ttgtgccaatactgatgaatcc	31
DXS1	ttgggatatggctcagcagt	cgcgggattctagcacaa	89
DXS2	gcaccaggaccagttctaattc	gcattttatcgtctgctgctt	4
GGPPS3	tgtcacaaagtcttctcagcaat	aagttaccttatcagcaaccaagtc	33
HDR	gtcgagcaaaagctggatct	tcctgtagatgtgaagtgttgctt	119
IDI	ttctctccgttgtcgtttcc	tcagaaacagcatcagccata	70
MCS	atgcaattttgggagctctg	cccttccatttaggatcagtgt	113
SDS	ggaaaggagactgttcaccaa	gtaccaggacgactgtgcaa	20
SIDPS	tgcggatgaattatcccttc	gggacctcagcaactaccat	111
