# Supplementary figures and images for: Environmental and Genetic Factors Associated with Solanesol Accumulation in Potato Leaves
Source: Front Plant Sci. 2016 Aug 25;7:1263. doi: 10.3389/fpls.2016.01263 (PMC4996988; doi:10.3389/fpls.2016.01263)

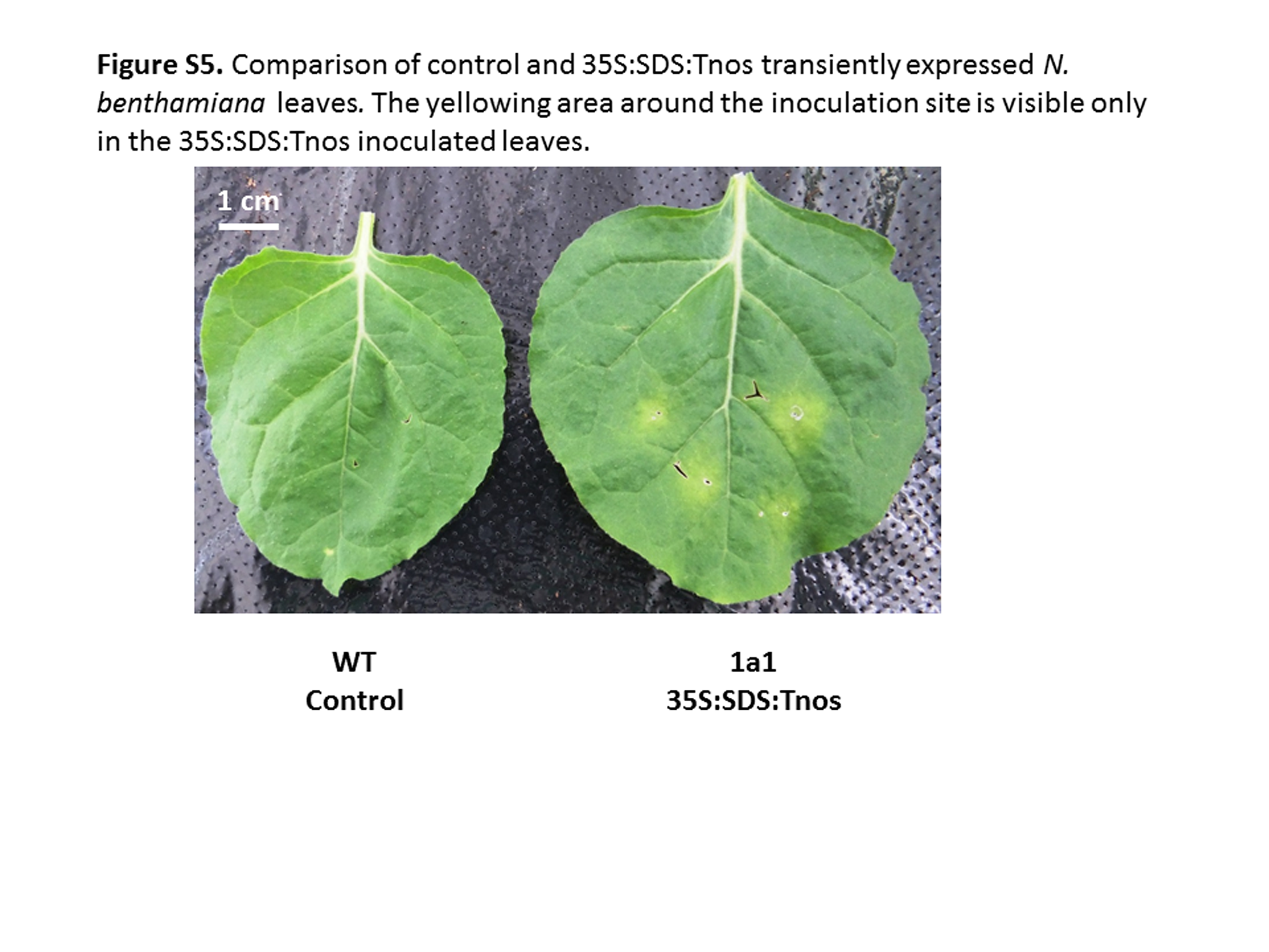

Supplement: Supplementary file 9 [file Image5.TIF]
